# Molecular Mechanisms of Spawning Habits for the Adaptive Radiation of Endemic East Asian Cyprinid Fishes

**DOI:** 10.34133/2022/9827986

**Published:** 2022-09-12

**Authors:** Feng Chen, Yeke Wang, Jun He, Liang Chen, Ge Xue, Yan Zhao, Yanghui Peng, Carl Smith, Jia Zhang, Jun Chen, Ping Xie

**Affiliations:** ^1^Donghu Experimental Station of Lake Ecosystems, State Key Laboratory of Freshwater Ecology and Biotechnology, Institute of Hydrobiology, Chinese Academy of Sciences, Wuhan 430072, China; ^2^University of Chinese Academy of Sciences, Beijing 100049, China; ^3^Department of Ecology and Vertebrate Zoology, University of Łódź, Łódź, Poland; ^4^Institute of Vertebrate Biology, Academy of Sciences of the Czech Republic, Brno, Czech Republic; ^5^Institute of Ecological Research and Pollution Control of Plateau Lakes, School of Ecology and Environment, Yunnan University, Kunming 650500, China

## Abstract

Despite the widespread recognition of adaptive radiation as a driver of speciation, the mechanisms by which natural selection generates new species are incompletely understood. The evolutionary radiation of endemic East Asian cyprinids has been proposed as evolving through a change in spawning habits, involving a transition from semibuoyant eggs to adhesive eggs in response to crosslinked river-lake system formation. Here, we investigated the molecular mechanisms that underpin this radiation, associated with egg hydration and adhesiveness. We demonstrated that semibuoyant eggs enhance hydration by increasing the degradation of yolk protein and accumulation of Ca^2+^ and Mg^2+^ ions, while adhesive eggs improve adhesiveness and hardness of the egg envelope by producing an adhesive layer and a unique 4th layer to the egg envelope. Based on multiomics analyses and verification tests, we showed that during the process of adaptive radiation, adhesive eggs downregulated the “vitellogenin degradation pathway,” “zinc metalloprotease pathway,” and “ubiquitin-proteasome pathway” and the pathways of Ca^2+^ and Mg^2+^ active transport to reduce their hydration. At the same time, adhesive eggs upregulated the crosslinks of microfilament-associated proteins and adhesive-related proteins, the hardening-related proteins of the egg envelope, and the biosynthesis of glycosaminoglycan in the ovary to generate adhesiveness. These findings illustrate the novel molecular mechanisms associated with hydration and adhesiveness of freshwater fish eggs and identify critical molecular mechanisms involved in the adaptive radiation of endemic East Asian cyprinids. We propose that these key egg attributes may function as “magic traits” in this adaptive radiation.

## 1. Introduction

Adaptive radiations represent rapid branching in the tree of life and are recognised as critical drivers of biodiversity. Studies of adaptive radiation have been central in developing our understanding of the mechanisms that drive speciation, diversification, and many associated ecological and evolutionary processes [1–4]. During an adaptive radiation, traits related to ecological and reproductive isolation play a major role in shaping biodiversity; examples include wing patterns in *Heliconius* butterflies [5], beak size and shape in Darwin's finches [6], and body and jaw shapes in African cichlid fishes [7]. Although the molecular mechanisms of some of these ecological traits have been identified, many remain incompletely understood.

Ecological speciation is one of the principal mechanisms by which natural selection generates new species [8, 9]. It occurs when adaptations to different environments or resources result in reproductive isolation. Ecological speciation is a major focus of evolutionary research and increasingly seen as a key driver of biodiversity [9, 10]. Ecological speciation is facilitated by traits that undergo divergent selection while also contributing to nonrandom mating. Termed “magic traits,” these may be the key to understanding how ecological speciation can be accomplished despite on-going, limited gene flow. Here, an underlying set of genes simultaneously controls both divergent adaptation and nonrandom mating [11, 12], potentially driving rapid diversification [13].

The endemic East Asian cyprinids, referred to as Xenocyprididae (Teleostei: Ostariophysi: Cypriniformes), are a natural assemblage of freshwater fishes found in eastern Asia, especially in China. This group includes approximately 44 genera and 151 species [14], which show diverse spawning habits that reflect adaptations to different flow conditions. Some species produce semibuoyant eggs in rivers during the rainy season, while others lay adhesive eggs that typically adhere to aquatic vegetation in lakes. A few fish also produce demersal eggs in slow-flowing streams. Chen et al. [15] showed that the spawning habits of this group evolved from a transition from producing semibuoyant eggs to adhesive eggs by approximately 13 Ma (Figure S1), coinciding with a peak in the net diversification rate of this endemic clade and a high intensity of the East Asian summer monsoon (Figure S2). These findings indicated that the rapid diversification of endemic East Asian cyprinids appeared to have been driven by variation in habits of spawning during the middle Miocene, which was closely linked to crosslinked river-lake system formation [15]. However, the molecular mechanisms underpinning the marked changes in spawning habits in this adaptive radiation remain unexplored.

**Figure 1:**
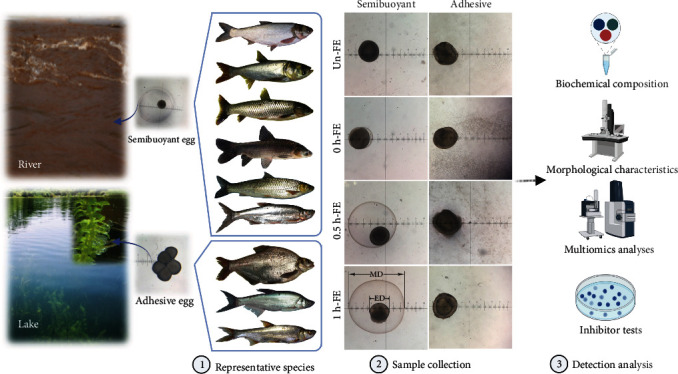
A schematic of the habitats associated with semibuoyant and adhesive eggs and the experimental design and data collection. Six representative species (*H. molitrix*, *H. nobilis*, *C. idella*, *M. piceus*, *S. curriculus*, and *C. alburnus*-B) of the endemic East Asian cyprinid group that produces semibuoyant eggs in lotic (rivers) habitats and three representative species (*M. amblycephala*, *C. dabryi*, and *C. alburnus*-A) of the endemic group that spawn adhesive eggs in lentic (lakes) habitats. Semibuoyant eggs and adhesive eggs were collected unfertilized (Un-FE) and 0, 0.5, and 1 h post fertilization (0 h-FE, 0.5 h-FE, and 1 h-FE) and used for elucidating the mechanisms of hydration and adhesiveness. MD: membrane diameter; ED: egg diameter.

Different spawning habits exhibit their own typical characteristics and mechanisms in response to different selection pressures. Semibuoyant eggs hydrate after ovulation and fertilization, absorbing water and expanding briefly to form a large perivitelline space, representing an adaptation to the flowing waters of large rivers (Figure 1). Although their density is slightly heavier than that of freshwater [16], semibuoyant eggs become suspended in the water column at river flow rates of 0.63–1.83 m/s [17], which facilitates dispersal as well as completion of hatching and development into young stages capable of independent movement [18]. The accumulation of lipid droplets in freshwater eggs is an alternative tactic to achieve buoyancy in lentic habitats; however, semibuoyant eggs in Cypriniformes typically contain almost no lipid droplets [16]. Marine pelagic eggs also take up large amounts of water in the oocytes during meiotic maturation and float in seawater after ovulation, a process that is associated with changes in osmolality and is regulated by free amino acids (FAAs) and ions and mediated by aquaporins [19–22]. While the mechanism for utilizing FAA derived from vitellogenin degradation is understood in marine pelagic eggs, accumulation of K^+^ and Na^+^ ions in the oocytes of demersal eggs, as well as K^+^ and Cl^−^ in the oocytes of pelagic eggs, might be regulated by the Na^+^, K^+^-ATPase, and Na^+^-K^+^-2Cl^−^ cotransport system and gap junctions [23–25]. However, the hydration of semibuoyant eggs in freshwater fish occurs *in vitro* in a hypotonic environment and the mechanisms involved remain opaque.

Adhesive freshwater fish eggs show an increase in adhesiveness after fertilization permitting them to adhere to aquatic plants, gravel, or other hard surfaces, which represents an adaptation to lentic environments (Figure 1). The degree of egg adhesiveness and the structure of the egg envelope are believed to reflect specific adaptations to particular environmental conditions related to the mode of reproduction [26]. Glycoproteins and mucopolysaccharide (glycosaminoglycan (GAG)) are reported to contribute to the development of egg stickiness [27–29]. In addition, the fish egg envelope (also referred to as the chorion or zona radiata) consisting of 2–4 zona pellucida (ZP) proteins varies in thickness, structure, and number of layers between or within species [30, 31], which may serve in protecting the embryo from physical and environmental stressors [32, 33]. However, the molecular mechanisms underlying the adhesiveness of freshwater eggs are similarly unknown. Since we know little about the molecular mechanisms of hydration and adhesiveness in freshwater fish eggs, understanding the evolutionary mechanisms associated with the evolutionary transition of adhesive eggs from semibuoyant eggs in the endemic East Asian cyprinids represents a key question in understanding this adaptive radiation.

Comparisons between individuals with different spawning habits within the same species facilitates the identification of changes specifically related to the evolution of egg type [34] and avoiding the interspecific interferences in metabolomic or proteomic analyses [35]. Notably, the topmouth culter (*Culter alburnus*) has two ecotypes: one (*C. alburnus-*A) lays adhesive eggs in lakes such as Lake Taihu, China, while the other (*C. alburnus*-B) spawns semibuoyant eggs in fast-flowing rivers such as the River Yangtze [36]. Consequently, *C. alburnus* offers an ideal model for investigating the molecular mechanisms of spawning habits underlying the adaptative radiation of endemic East Asian cyprinids.

In this study, we compared the semibuoyant eggs of six representative species (*C. alburnus*-B, *Hypophthalmichthys molitrix*, *H. nobilis*, *Ctenopharyngodon idella*, *Mylopharyngodon piceus*, and *Squaliobarbus curriculus*), with the adhesive eggs of further three representative species (*C. alburnus*-A, *Megalobrama amblycephala*, and *C. dabryi*) belonging to the endemic East Asian cyprinid group (Figure 1), demonstrating consistent changes in the biochemical composition and morphological characteristics of the eggs with the same spawning habit. Based on histochemical staining, multiomics analyses, and immunofluorescence in the eggs of the two spawning habits of *C. alburnus* in combination with inhibitor tests of zebrafish eggs, we aimed to illustrate the molecular mechanisms associated with hydration and adhesiveness in freshwater fish eggs and thereby elucidate the potential mechanism for the evolution of adhesive eggs from semibuoyant eggs in the adaptive radiation of endemic East Asian cyprinids.

## 2. Results

### 2.1. Biochemical Composition

#### 2.1.1. Water Content and Osmotic Gradient Experiment

**Figure 2:**
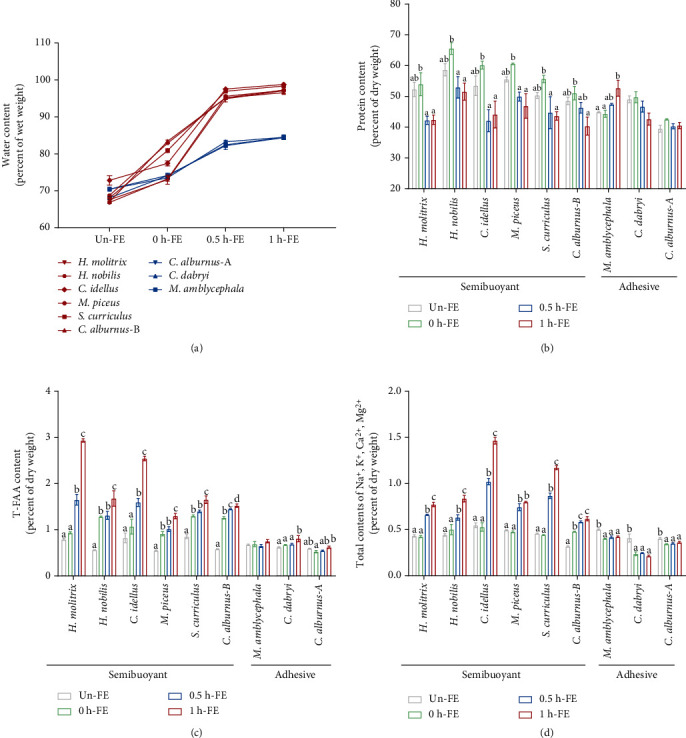
Contrasting spawning habits differ in their biochemical composition of unfertilized eggs (Un-FE) and 0, 0.5, and 1 h post fertilization (0 h-FE, 0.5 h-FE, and 1 h-FE). (a) The water contents of semibuoyant eggs (red lines) and adhesive eggs (blue lines). (b) The protein contents, (c) T-FAA contents, and (d) the total contents of Na^+^, K^+^, Ca^2+^, and Mg^2+^ ions of different spawning habits. Values are presented as the mean ± SEM from three to five separate experiments. Different lowercase letters indicate significant differences among the four time periods (*p* < 0.05, one-way analysis of variance). T-FAA: total free amino acid.

The water contents of semibuoyant eggs were higher than those of adhesive eggs 0.5 and 1 h after fertilization (Figure 2(a)), and the perivitelline space volumes (Vm/Ve) of semibuoyant eggs were similarly greater than those of adhesive eggs 0.5 and 1 h after fertilization (Figure S3). An osmotic gradient experiment performed on eggs 1 h after fertilization showed that the membrane diameter changes (1 h-FE MD/0 h-FE MD) observed in semibuoyant eggs, which showed an increase in the osmotic concentration of the solution, were significantly reduced and irregular in adhesive eggs (Figure S4).

#### 2.1.2. Protein, Total Free Amino Acid, and Ion Contents

The protein contents of semibuoyant eggs decreased after fertilization, while they were irregular in adhesive eggs (Figure 2(b)). After fertilization, the T-FAA contents of semibuoyant eggs increased over time compared with those of adhesive eggs (Figure 2(c)). The total contents of Na^+^, K^+^, Ca^2+^, and Mg^2+^ ions were also markedly increased in semibuoyant eggs but reduced in adhesive eggs (Figure 2(d)). The major contents of Ca^2+^ and Mg^2+^ ions were conspicuously higher in semibuoyant eggs after fertilization relative to adhesive eggs, while the contents of Na^+^ and K^+^ ions were irregular in both semibuoyant and adhesive eggs (Figure S5). The effect of the T-FAA contents on osmolality was considerably lower than that of the ion contents of freshwater semibuoyant eggs (Table S1).

### 2.2. Morphological Characteristics of the Egg Envelope

**Figure 3:**
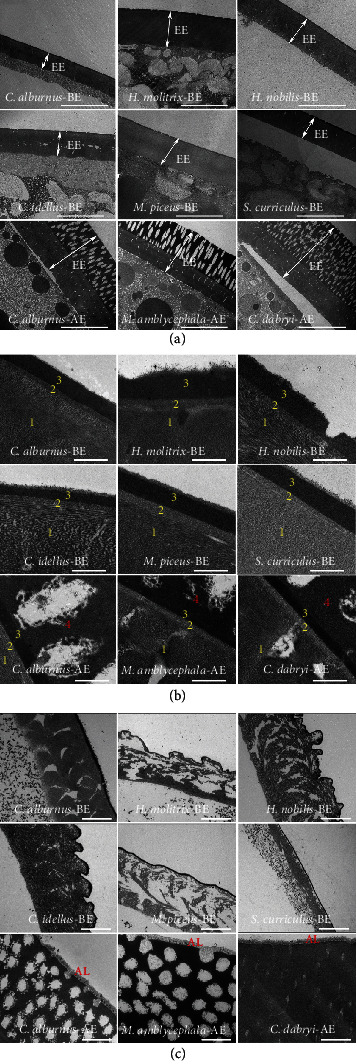
The egg envelope ultrastructure of six representative species with semibuoyant eggs (*C. alburnus*-B, *H. molitrix*, *H. nobilis*, *C. idella*, *M. piceus*, and *S. curriculus*) and three representative species with adhesive eggs (*C. alburnus*-A, *M. amblycephala*, and *C. dabryi*) of endemic East Asian cyprinids. (a) TEM images of the egg envelopes of unfertilized eggs (scale bar is 10 *μ*m). EE: egg envelope. (b) TEM images of the egg envelopes of unfertilized eggs (scale bar is 500 nm). The egg envelopes of semibuoyant eggs were divided into three layers (1, 2, and 3), and the egg envelopes of adhesive eggs were divided into four layers (1, 2, 3, and 4). (c) TEM images of the adhesive layer of eggs at 1 h post fertilization (scale bar is 2 *μ*m). AL: adhesive layer; BE: semibuoyant egg; AE: adhesive egg.

Microstructure analysis by transmission electron microscopy (TEM) of ovulated mature eggs and fertilized eggs 1 h post fertilization showed that the egg envelopes of semibuoyant eggs were divided into three layers (Figure 3(b)), while the egg envelopes of adhesive eggs were further divided into four layers (Figure 3(b)). Adhesive eggs possessed an adhesive layer (AL) 1 h after fertilization, while semibuoyant eggs did not develop an AL (Figure 3(c)).

### 2.3. Histochemical Staining and Monosaccharide Composition

**Figure 4:**
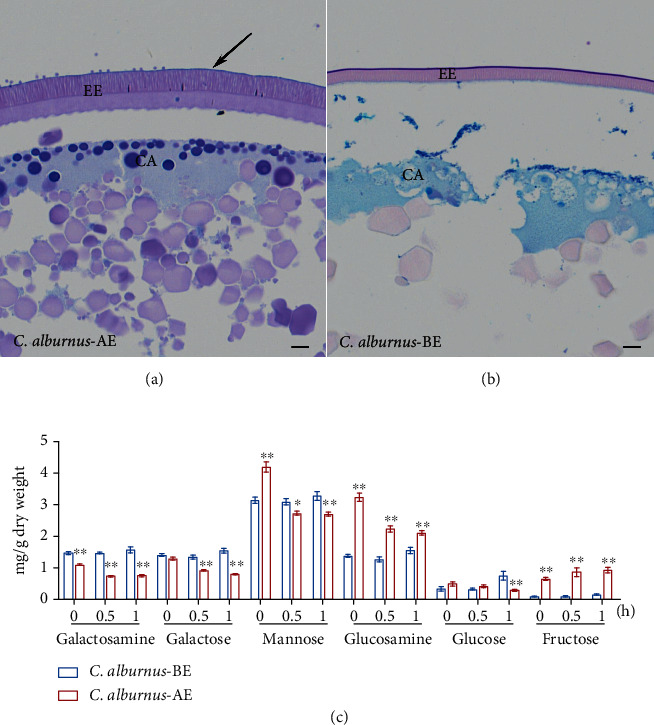
Histological characterization and monosaccharides of the egg envelope in two spawning habits of *C. alburnus* eggs after fertilization. (a, b) Histological characterization staining with Alcian blue- (pH 2.5) periodic acid Schiff (AB-PAS) in the adhesive and semibuoyant eggs of *C. alburnus* at 1 h after fertilization (scale bar is 10 *μ*m). Black arrow: outer edge of the 4th layer of the adhesive egg envelope; AE: adhesive egg; BE: semibuoyant egg; EE: egg envelope; CA: cortical alveoli. (c) The monosaccharide analysis of the egg envelopes of the two types of *C. alburnus* eggs was performed at 0, 0.5, and 1 h after fertilization. Values are presented as the mean ± SEM. ^∗^indicates *p* < 0.05 versus *C. alburnus*-BE and ^∗∗^indicates *p* < 0.01 versus *C. alburnus*-BE.

Histochemical staining of eggs in *C. alburnus* 1 h after fertilization showed that the surface of the egg envelope and cortical area in adhesive eggs of *C. alburnus*-A, as well as the cortical area in semibuoyant eggs of *C. alburnus*-B, produced positive reactions (blue-violet) (Figure 4). Six monosaccharides were detected in the egg envelope of *C. alburnus* at 0, 0.5, and 1 h post fertilization. The glucosamine and fructose contents of the egg envelopes of *C. alburnus*-A following fertilization were significantly higher than those in *C. alburnus-*B, while the galactosamine and galactose contents in the egg envelopes of *C. alburnus*-A after fertilization were significantly lower than those of *C. alburnus-*B (Figure 4).

### 2.4. Proteome Analysis

Among the total identified proteins in the eggs and ovaries of *C. alburnus*, 7017 proteins could be quantified using tandem mass tag (TMT) labelling and high-performance liquid chromatography (HPLC) fractionation followed by tandem mass spectrometry (MS/MS) analysis. Among the quantified proteins, 614, 530, 728, 902, and 1434 differentially expressed proteins (DEPs) between two spawning habits were identified in the eggs at unfertilized, 0, 0.5, and 1 h post fertilization and ovaries. DEPs of pathways of yolk protein degradation, including (i) the “vitellogenin (Vtg) degradation pathway,” (ii) the “zinc metalloproteinase pathway,” and (iii) the “ubiquitin-proteasome pathway,” Ca^2+^ and Mg^2+^ active transport, and the vitelline envelope permeability transition pore in the eggs were involved in regulating the mechanism of hydration (Table S2). DEPs related to the crosslinks of microfilament and adhesiveness in adhesive eggs were upregulated compared to those in semibuoyant eggs. In contrast to the *C. alburnus-*B ovary, the protein expression levels of Gfpt1, Gpi1, Nagk, and Udgh were significantly upregulated in the *C. alburnus-*A ovary (Table S3).

Egg envelope proteins of *C. alburnus* were identified, among which 1714 proteins could be quantified through label-free HPLC fractionation followed by LC-MS/MS analysis. Among these proteins, 360 DEPs between two spawning habits were identified in the egg envelopes at 1 h post fertilization. DEPs associated with the crosslinks of microfilament and adhesiveness in adhesive egg envelopes were remarkably upregulated in comparison with those in semibuoyant egg envelopes (Table S4). Compared to semibuoyant egg envelopes, the protein expression levels of ZP2 and ZP3X1 involved in the hardening and adhesiveness of the egg envelope were significantly upregulated in adhesive egg envelopes.

### 2.5. Transcriptome Sequence Analysis, Assembly, and Functional Annotation

Pooled cDNA libraries derived from eggs and ovarian tissues of *C. alburnus* were prepared and sequenced (Table S5), and the total number of unigenes ranged from 123 to 17562 bp, with an average length of 2424 bp and an N50 of 2981 bp. The length distribution of all transcripts was shown in Figure S6. Annotation results of Venn diagram analysis and the statistical results of the GO and KEGG classification of all annotated unigenes were shown in Figure S7. Compared with the *C. alburnus*-B ovary, the mRNA expressions of enzymes involved in biosynthesis of heparan sulphate (HS) and chondroitin sulphate (CS)/dermatin sulphate (DS) were significantly altered in the *C. alburnus*-A ovary (Table S6).

### 2.6. Immunofluorescence

**Figure 5:**
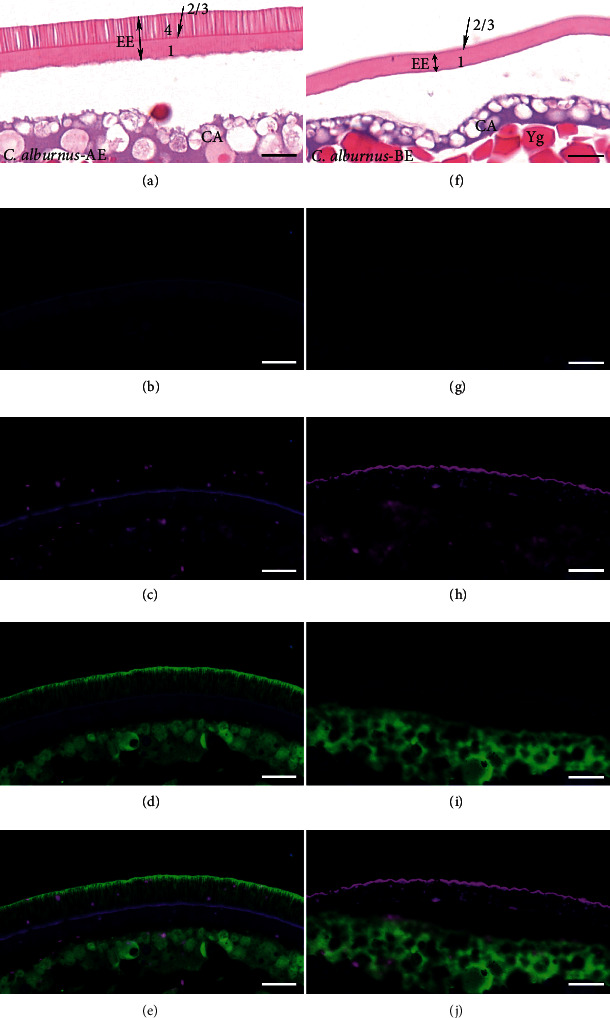
Co-immunofluorescence analysis demonstrating localization of egg envelope proteins (ZP2 and ZP3X1) in unfertilized eggs (scale bar is 20 *μ*m). (a–e) Adhesive egg of *C. alburnus* (*C. alburnus*-AE); (f–j) semibuoyant egg of *C. alburnus* (*C. alburnus*-BE); (a, f) stained results with haematoxylin-eosin; (b, g) control groups; (c–e and h–j) immunofluorescence localization of ZP2 (magenta) and ZP3X1 (green) in the egg envelope. EE: egg envelope; CA: cortical alveoli; Yg: yolk globule.

Based on the immunofluorescence localization analysis, we verified the expression of the egg envelope proteins ZP2 and ZP3X1, which were differentially expressed proteins in the egg envelope proteomics (Table S4). The immunofluorescence results showed that ZP2 was located in two spawning habits of egg envelopes (the 2nd/3rd layer), while ZP3X1 was located in the adhesive egg envelope (the 4th layer) (Figure 5).

### 2.7. Inhibitor Tests

**Figure 6:**
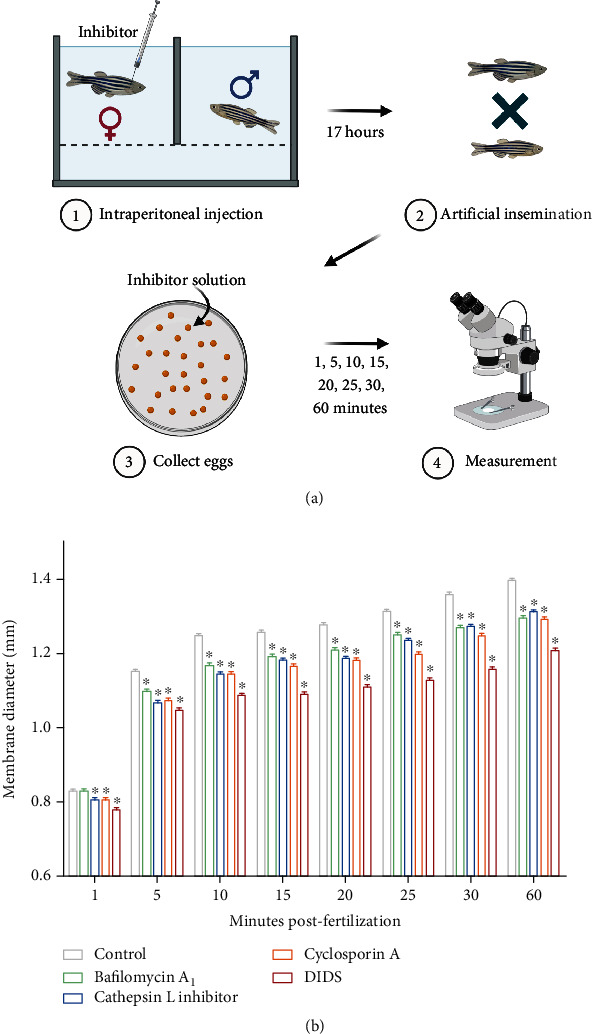
A scheme for the experimental design and the membrane diameter of zebrafish eggs after inhibition. (a) Mature female zebrafish were treated with 0.05% DMSO, 100 nM bafilomycin A_1_, 1.5 *μ*M cathepsin L inhibitor, 5 *μ*M cyclosporine A, and 200 *μ*M DIDS by intraperitoneal injection (*n* = 3). After artificial insemination, zebrafish eggs were immersed in DMSO or inhibitor solutions and their membrane diameter was measured at 1, 5, 10, 15, 20, 25, 30, and 60 minutes post fertilization. (b) The membrane diameter of zebrafish eggs at 1, 5, 10, 15, 20, 25, 30, and 60 minutes post fertilization when exposed to DMSO or inhibitor solutions. Values are presented as the mean ± SEM from three separate experiments (30–40 per group in each experiment). Asterisks indicate significant differences between the control and treatment groups (*p* < 0.05, Student's *t*-test). DMSO: dimethyl sulfoxide; DIDS: 4,4′-diisothiocyanatostilbene-2,2′-disulfonic acid disodium salt.

We used solutions of bafilomycin A_1_, cathepsin L inhibitor, cyclosporine A, and 4,4′-diisothiocyanatostilbene-2,2′-disulfonic acid disodium salt (DIDS) to inhibit the activities of V-ATPase, cathepsin L, cyclophilin D, and voltage-dependent anion-selective channel protein in zebrafish eggs, respectively. We found that the membrane diameters of zebrafish eggs at 1, 5, 10, 15, 20, 25, 30, and 60 min post fertilization were significantly reduced by bafilomycin A_1_, cathepsin L inhibitor, cyclosporine A, and DIDS relative to those in controls (Figure 6).

## 3. Discussion

### 3.1. Biochemical Changes for Hydration in Freshwater Fish Eggs

Our results show that semibuoyant eggs undergo significant hydration after they are produced, with absorbed water causing expansion of the perivitelline space. The hydration of semibuoyant eggs results in their suspension in the water column in fast-flowing rivers [16]. Marine pelagic eggs complete hydration in the ovary to adapt to the hyperosmotic environment after ovulation [19–22]. In contrast, adhesive eggs attach to aquatic plants, gravel, or other hard surfaces where they complete the process of hatching, a process that is not achieved through hydration.

Based on the osmotic gradient of eggs, we found that osmotic pressure was the major factor regulating the hydration of freshwater fish eggs, especially semibuoyant eggs. This phenomenon was reported in marine pelagic eggs [37–39] but rarely described in freshwater semibuoyant eggs. The T-FAA and ion contents of fish eggs are two major components of osmolality. In the present study, the increased T-FAA contents and total contents of Na^+^, K^+^, Ca^2+^, and Mg^2+^ ions of semibuoyant eggs with time after fertilization suggested that freshwater fish eggs achieved hydration through an increased T-FAA and ion contents. Although marine pelagic eggs achieve hydration in the ovary in the same way [37, 40, 41], the effect of the T-FAA content on the osmolality of marine pelagic eggs is considerably greater than that of freshwater semibuoyant eggs. This result indicates that fishes have likely evolved divergent metabolic pathways under different salinity regimes.

### 3.2. Mechanisms Underlying the Hydration of Semibuoyant Eggs

#### 3.2.1. Three Pathways of Yolk Protein Degradation

**Figure 7:**
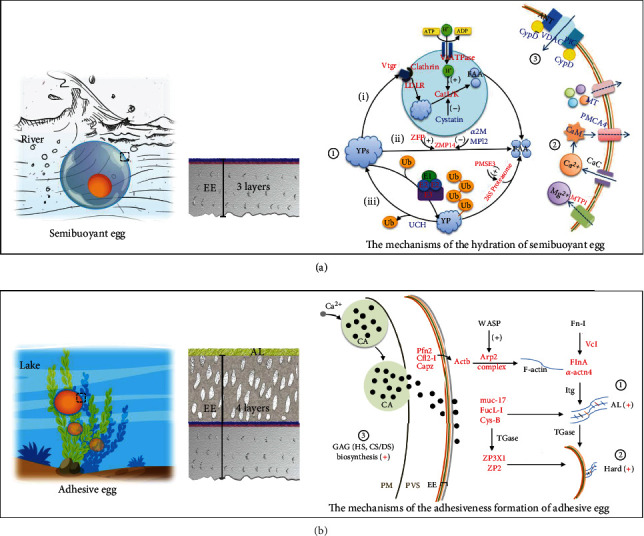
A schematic summary of the molecular mechanisms of two spawning habits for the adaptive radiation of endemic East Asian cyprinids. (a) The mechanisms underlying the hydration of semibuoyant eggs. ① Three pathways of yolk protein degradation. (i) Vtg degradation pathway, (ii) zinc metalloproteinase pathway, and (iii) ubiquitin-proteasome pathway. ② The pathways of Ca^2+^ and Mg^2+^ active transport. ③ The molecular structure of the egg envelope permeability transition pore. Proteins in red and blue show significant upregulation and downregulation in semibuoyant eggs, respectively. (b) The mechanisms underlying adhesiveness formation of adhesive eggs. ① The crosslinks of microfilament-associated proteins and adhesive-related proteins. ② The hardening of the egg envelope. Proteins in red show significant upregulation in adhesive eggs. Details are shown in Tables S2 and S4. ③ The biosynthesis of glycosaminoglycan (GAG) in the ovary, including the biosynthesis of immediate precursors of GAG determined by proteomics analysis (details are shown in Table S3 and Figure S8) and the synthesis and modification of heparan sulphate (HS) and chondroitin/dermatan sulphates (CS/DS) by transcriptomics analysis (details are shown in Table S6 and Figure 8). EE: egg envelope; PVS: perivitelline space; Yp: yolk protein; FAA: free amino acids; Vtg: vitellogenin; Ub: ubiquitin; CaC: Ca^2+^ channel; CA: cortical alveoli; PM: plasma membrane; AL: adhesive layer.

In our study, the decreased protein contents and increased T-FAA levels resulted from yolk protein degradation, which might be involved in regulating the process of hydration in semibuoyant eggs. Proteomic analyses suggested that three pathways of yolk protein degradation might regulate the mechanisms of hydration in semibuoyant eggs (Figure 7(a); Table S2). In contrast to adhesive eggs, semibuoyant eggs enhanced the vitellogenin receptor, clathrin, and low-density lipoprotein receptor to induce vitellogenin endocytosis and increased V-ATPase and decreased cystatin to guide cathepsin toward vitellogenin degradation. As vitellogenin is the main component of yolk protein, the “Vtg degradation pathway” is a major mechanism of yolk protein degradation in freshwater semibuoyant eggs, similar to that of marine pelagic eggs [20–22]. However, the “zinc metalloproteinase pathway” and “ubiquitin-proteasome pathway,” which were filtered from the proteomic results, might also be involved in yolk protein degradation in freshwater semibuoyant eggs, which has not been reported for marine pelagic eggs. In the “zinc metalloproteinase pathway,” semibuoyant eggs increased the zinc finger protein and reduced *α*-2-macroglobulin-like and metalloproteinase inhibitor 2-like to improve yolk protein degradation of zinc metalloproteinase nas-14-like in comparison with adhesive eggs (Figure 7(a); Table S2). Moreover, compared to adhesive eggs, semibuoyant eggs enhanced ubiquitin-like 1-activating enzyme, ubiquitin-conjugating enzyme, and ubiquitin-protein ligase of ubiquitination and decreased ubiquitin carboxyl-terminal hydrolase of deubiquitination, while increasing proteasome activator complex subunit 3 to elevate yolk protein degradation of 26S proteasome non-ATPase regulation in the “ubiquitin-proteasome pathway” (Figure 7(a); Table S2). In our inhibitor tests, the membrane diameters of zebrafish eggs were shown to be significantly reduced by bafilomycin A_1_ and cathepsin L inhibitor treatment relative to those of the control, suggesting that V-ATPase and cathepsin L affect the hydration of freshwater fish eggs.

#### 3.2.2. Pathways of Ca^2+^ and Mg^2+^ Active Transport

Our results indicated that semibuoyant eggs took up more Ca^2+^ and Mg^2+^ ions to increase their hydration. In seawater, the cationic osmolality of pelagic eggs was determined by K^+^ and Na^+^ [19, 37, 42]. This difference is probably related to the greater concentration of Ca^2+^ and Mg^2+^ ions in freshwater (the concentrations of Ca^2+^, Mg^2+^, K^+^, and Na^+^ ions are typically 44.72 ± 8.41, 23.10 ± 1.73, 9.53 ± 1.52, and 20.27 ± 2.12 mg · L^−1^, respectively), with more Na^+^ and K^+^ ions in marine fish ovarian fluid (the concentrations of Na^+^, K^+^, and Ca^2+^ ions in ovarian fluid of *Scophthalmus maximus* are 198–218, 7.4–15.0, and 2.3–4.2 mM, respectively [43]). The active transport pathways of Ca^2+^ and Mg^2+^ were significantly enhanced in semibuoyant eggs (Figure 7(a); Table S2). Compared with adhesive eggs, semibuoyant eggs showed increased magnesium transport protein 1, decreased calmodulin-like and plasma membrane calcium ATPase 4 to restrain calcium discharge, and decreased metallothionein to repress divalent cation efflux in the active transport pathway. These results indicated that semibuoyant eggs expressed greater Mg^2+^ influx and reduced Ca^2+^ and Mg^2+^ efflux which increased osmolality and hydration.

#### 3.2.3. The Egg Envelope Permeability Transition Pore

The permeability transition pore is pervious to solutes smaller than 1.5 kDa, such as ions, and is composed of cyclophilin D (CypD), voltage-dependent anion-selective channel protein (VDAC), adenine nucleotide transporter (ANT), and phosphate carrier protein (PiC) in the vitelline envelope [44–46], which might be essential for ion transport and water permeability. Proteomic analyses indicated that the protein expression levels of CypD, VDAC, and PiC were significantly lower in semibuoyant eggs than in adhesive eggs (Figure 7(a); Table S2). In the inhibitor tests, the membrane diameters of zebrafish eggs were significantly reduced by cyclosporine A and DIDS relative to those in controls, which indicates that CypD and VDAC could regulate the hydration of freshwater fish eggs. In seawater, the water permeability of pelagic eggs depends on aquaporins [47], which we did not detect in the proteomic results of *C. alburnus* eggs and egg envelopes.

Taken together, our results indicate that semibuoyant eggs increase osmotic pressure by accumulating ions and degrading yolk proteins resulting in water absorption and the formation of a large perivitelline space. In flowing water, the high level of hydration causes eggs to be suspended in the water column thereby facilitating dispersal while avoiding damaging abrasion on the river bed [16]. Since the osmolality of semibuoyant eggs was approximately 30-fold higher than that of freshwater, the hydration of semibuoyant eggs was easily achieved and did not depend primarily on yolk protein degradation. In a hyperosmotic environment, marine pelagic eggs take up water during oocyte maturation in the ovary, where hydration is more difficult to achieve, and an alternative mechanism based predominantly on degradation of vitellogenin permits an increase in osmotic pressure and hydration by aquaporin, thereby enabling eggs to achieve neutral or positive buoyancy. Consequently, marine pelagic eggs and freshwater semibuoyant eggs appear to have evolved alternative mechanisms of hydration under different environmental pressures.

### 3.3. Mechanisms Underlying the Adhesiveness of Adhesive Eggs

#### 3.3.1. The Hardening of the Egg Envelope

The consequences of morphological divergence in lacustrine species likely facilitated their expansion into lake ecosystems. Adhesive eggs, with the egg envelope possessing an AL, permit attachment to submerged vegetation and structures, which is critical for egg survival in lentic environments to prevent them from being suffocated by anoxic sediment [39]. We demonstrated that adhesive eggs possess a thick envelope, including four layers, with the 4th layer contributing to resistance to environmental stressors to which eggs are exposed on a substrate. ZP2 and ZP3X1, which are components of ZP proteins, were significantly upregulated in adhesive egg envelopes; ZP2 was located in the 2nd/3rd layer of the egg envelope in both two egg types, while ZP3X1 was only located in the 4th layer of the adhesive egg envelope. ZP2 could be crosslinked with ZP3 by the *γ*-glutamyl-*ε*-lysine isopeptide catalysed by transglutaminase (TGase), which is associated with fish egg envelope hardening [31, 48, 49]. TGase involved in the crosslinking of ZPs was identified as enhanced in adhesive eggs (Table S2). Taken together, it seems that adhesive eggs undergo egg envelope hardening, which toughens an otherwise delicate structure following fertilization, thereby resisting stressors to which eggs are exposed in benthic environments.

#### 3.3.2. The Crosslinks of Microfilament-Associated Proteins and Adhesive-Related Proteins

Multiple microfilament-associated proteins were clearly upregulated in the adhesive egg envelope of *C. alburnus*, including actin, Arp 2, filamin-A, *α*-actinin, vinculin, profilin, cofilin, and capping protein (Tables S2 and S4). The Arp 2/3 complex, activated by WASP proteins, is involved in mediating actin polymerization and inducing the initiation of new filaments [50, 51]. Filamin-A and *α*-actinin stabilize the entire network by crosslinking actin filaments [50]. Vinculin and talin, which bind to fibronectins, form a network with actin filaments and may be beneficial to fish egg morphology [52], while profilin, cofilin, and capping protein are involved in restricting polymerization to new filaments that are close to the plasma membrane [50, 51, 53]. Fibronectin, collagen, actin, and myosin are also substrates of TGase [54–56]. Moreover, three adhesion-related proteins (mucin, fucolectin, and cystatin-B) were upregulated in the adhesive egg envelope of *C. alburnus* (Table S4), implying that they might be functionally associated with egg adhesion [57–59]. Further recruitment of cystatins via the regulation of surface TGase is essential for cell adhesion [60, 61]. Thus, microfilament-associated proteins, adhesive-related proteins, and egg envelope proteins (ZPs) involved in crosslinking that is catalysed by TGase may function in enhancing egg envelope hardness and egg attachment strength in adhesive eggs (Figure 7(b)).

#### 3.3.3. The Biosynthesis of Glycosaminoglycan in the Ovary

**Figure 8:**
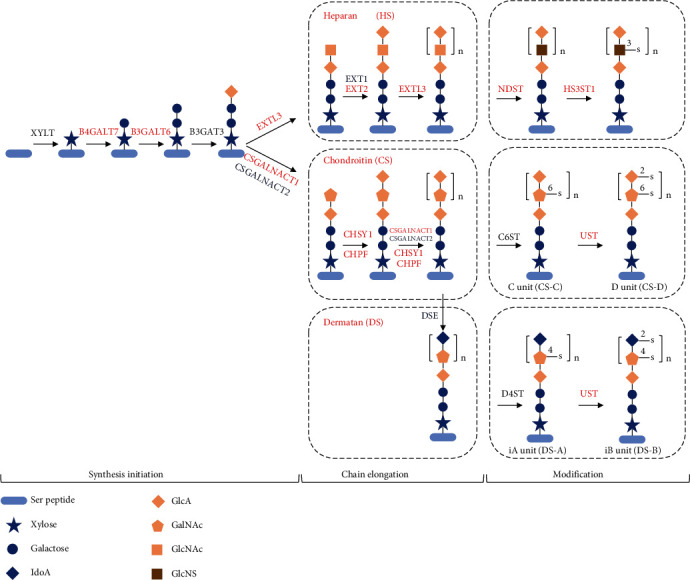
The transcription level of biosynthesis of glycosaminoglycan (GAG) in the ovary, including heparan sulphate (HS) and chondroitin/dermatan sulphate (CS/DS) core structures and modification. Genes in red involved in synthesis initiation, chain elongation, and modification in the biosynthesis of GAG are significantly upregulated in ovaries producing adhesive eggs. Details are shown in Table S6. Ser peptide: light blue ovals; xylose residue: blue star; galactose: blue circle; iduronic acid (IdoA): blue diamond; glucuronic acid (GlcA): orange diamond; N-acetylgalactosamine (GalNAc): orange pentagon; N-acetylglucosamine (GlcNAc): orange square; N-sulfoglucosamine (GlcNS): brown square. B4GALT7: *β*-1,4-galactosyltransferase 7; B3GALT6: *β*-1,3-galactosyltransferase 6; EXTL3: exostosin-like 3; EXT1: exostosin-1b; EXT2: exostosin-2; NDST: heparan sulphate N-deacetylase/N-sulfotransferase; HS3ST1: heparan sulphate glucosamine 3-O-sulfotransferase 1; CSGALNACT1: chondroitin sulphate N-acetylgalactosaminyltransferase 1; CSGALNACT2: chondroitin sulphate N-acetylgalactosaminyltransferase 2; CHSY1: chondroitin sulphate synthase 1; CHPF: chondroitin polymerizing factor a; UST: uronyl 2-sulfotransferase; DSE: dermatan-sulphate epimerase; D4ST: dermatan 4 sulfotransferase 1.

A positive reaction of Alcian blue- (pH 2.5) periodic acid Schiff (AB-PAS) staining indicated the presence of neutral glycoproteins and acid glycoconjugates on the fertilized adhesive egg envelopes comprising mucosubstances that may be associated with egg adhesiveness. This finding contrasted to that for semibuoyant eggs of *C. alburnus*. The relative protein expression levels of four enzymes (Gfpt1, Gpi1, Nagk, and Udgh) significantly upregulated in the *C. alburnus-*A ovary were involved in catalysing the early steps in GAG biosynthesis (Figure S8), which suggested that adequate precursor pools were present for the large increase in GAG biosynthesis during oogenesis in *C. alburnus*-A. The mRNA expression of some enzymes involved in GAG (HS, CS/DS) biosynthesis and modification was significantly changed in the *C. alburnus*-A ovary (Figure 8 and Table S6), which suggested that the biosynthesis and modification of GAG (HS and CS/DS) might be enhanced in *C. alburnus*-A. Glucosamine is one of the units of the repeating disaccharides of HS which is the preferred substrate for cell adhesion [62], and its content in the adhesive egg envelope of *C. alburnus*-A after fertilization was significantly higher than in *C. alburnus-*B. Consequently, the enhancement of GAG biosynthesis in *C. alburnus*-A might provide the basis for the development of adhesiveness, with GAG potentially interacting with the proteins of the egg envelope or adhesive layer (Figure 7(b)).

In this study, we showed consistent biochemical and morphological changes associated with the same spawning habit. Based on multiomics analyses, we demonstrated that semibuoyant eggs upregulated the “Vtg degradation pathway,” “zinc metalloprotease pathway,” and “ubiquitin-proteasome pathway” to improve yolk protein degradation and upregulated the accumulation of Ca^2+^ and Mg^2+^ ions passing through the vitelline envelope permeability transition pore to enhance their hydration in fast-flowing rivers, which contrasts with that seen in marine pelagic eggs from a hyperosmotic environment. Adhesive eggs were shown to upregulate the crosslinks of microfilament-associated proteins and adhesive-related proteins, the hardening-related proteins of the egg envelope, and the biosynthesis of GAG in the ovary to produce an adhesive layer and 4th layer of the egg envelope to improve the adhesiveness and hardness of the egg, while downregulating the pathways involved in hydration.

The diversification of spawning habits associated with the evolution of adhesive eggs from semibuoyant eggs among endemic East Asian cyprinids is associated with key adaptive changes relating to egg hydration and adhesiveness. We propose that these two key egg attributes may function as “magic traits” in the adaptive radiation of endemic East Asian cyprinids. Most research on magic traits has hitherto focused on traits contributing to male mating signals and female preference for these signals [4, 8]. While convincing in specific cases [11, 13], the current consensus is that magic traits are unlikely to be widespread. Thus, the novel molecular mechanisms associated with egg hydration and adhesiveness, which we have elucidated in this study, may represent new examples of “magic traits” that underpin the adaptive radiation of endemic East Asian cyprinids.

## 4. Materials and Methods

### 4.1. Sample Collection

Eggs of representative East Asian endemic cyprinids, including *Hypophthalmichthys molitrix*, *H. nobilis*, *Ctenopharyngodon idella*, *Mylopharyngodon piceus*, *Spualiobarbus curriculus*, *Culter alburnus-*B, *Megalobrama amblycephala*, *C. dabryi*, and *C. alburnus-*A, were collected by artificial propagation from fishery facilities in Jingzhou and Ezhou, Hubei province (in the River Yangtze Basin), from April to July, 2017, to 2019. Mature females and males were stimulated to spawn using the maturation-inducing steroid (MIS) 17*α*, 20*β*-dihydroxy-4-pregnen-3-one. Ovulated eggs were divided into four groups: unfertilized eggs and eggs at 0, 0.5, and 1 h postfertilization (Figure 1), which were maintained in freshwater (osmolality was 9.3 ± 1.2 mosmol · kg^−1^, and Mg^2+^, K^+^, Ca^2+^, and Na^+^ concentrations were 23.10 ± 1.73, 9.53 ± 1.52, 44.72 ± 8.41, and 20.27 ± 2.12 mg · L^−1^, respectively). One portion of the samples was placed in Bouin's solution or 2.5% glutaraldehyde, and the remaining eggs were immediately frozen in liquid nitrogen and stored at −80°C until analysis. Unfertilized or fertilized eggs were added to the barrel of a 1 or 2 ml syringe with a fine needle. The ooplasmic components of unfertilized eggs were removed with EDTA-saline five times [49], and the ooplasmic components of fertilized eggs were removed with pure water. After centrifugation at 3000 g for 1 min, the egg envelopes were immediately frozen in liquid nitrogen and stored at −80°C until analysis. All procedures were performed with the approval of the Animal Care and Use Committee of the Institute of Hydrobiology, Chinese Academy of Sciences (approval ID: IHB 20140724).

### 4.2. Water Content

The weighed eggs (wet mass, *W*_*m*_ (g) *n* = 3 to 5, 1–3 g per replicate) were lyophilized in a vacuum freeze drier (ALPHA 1-4, Christ, Germany). After 48 h, their dry mass (*W*_*d*_ (g)) was determined with a millibalance (BSA124S, Sartorius, Germany) and they were stored in a desiccator. The egg water content was calculated using the following equation: water content (%) = 100 (*W*_*m*_ − *W*_*d*_) *W*_*m*_^−1^.

### 4.3. Protein Content

Egg samples of 0.2–0.5 g per replicate (*n* = 3 or 4) were homogenized in radioimmunoprecipitation assay buffer (RIPA) (Beyotime Biotechnology, China) with 1 mM phenylmethanesulfonyl fluoride (PMSF) (Beyotime Biotechnology, China) for 2 min using a standard microhomogenizing package (PRO-PK-02200S, PRO Scientific, USA). The homogenate was centrifuged at 12000 g for 10 min at 4°C, and the supernatant was collected. The supernatant was diluted tenfold using RIPA buffer, and the protein content was determined with an Enhanced BCA Protein Assay Kit (Beyotime Biotechnology, China).

### 4.4. Free Amino Acid and Inorganic Ion Contents

Egg samples of 0.5–1 g per replicate (*n* = 3 or 4) were homogenized in 5 mL of 15 mM HCl for 3–5 min by using a standard microhomogenizing package. Samples were centrifuged at 12000 g for 10 min at 4°C, and the supernatant was collected. One millilitre of the supernatant was collected and mixed thoroughly with 1 mL of sulfosalicylic acid (Sinopharm Chemical Reagent Co. Ltd., China) for 1 h at 4°C. After centrifugation at 12000 g for 15 min at 4°C, the supernatant was collected and filtered through a 0.22 *μ*m sieve (Jinteng, China). One portion of the supernatant was then subjected to free amino acid measurement with an amino acid analyser (A300, MembraPure, Germany), and the remaining portion of the supernatant used for inorganic ion content measurement was diluted tenfold with deionized H_2_O, followed by measurement with an optical emission spectrometer (Optima 8000, PerkinElmer, USA).

### 4.5. Electron Microscopy

For TEM, fish eggs were prefixed in 2.5% glutaraldehyde solution and then fixed in 1% aqueous osmium tetroxide. The specimens were embedded in epon-araldite after dehydration. Ultrathin sections were cut with diamond knives on an EM UC7 ultramicrotome (Leica, Germany), treated with the contrast agents uranyl acetate and lead citrate and examined on a Tecnai G^2^ 20 TWIN microscope (FEI, USA).

### 4.6. Histological Analysis

For light microscopy, fish eggs were fixed in Bouin's solution and embedded in paraffin and blocks were sectioned on a microtome. The 5 *μ*m thick sections with the largest cut surface were stained with haematoxylin and eosin (H&E). AB-PAS staining was used to determine the histochemical contents of the egg structures. Histological observations were performed using light microscopy (Nikon Eclipse E100, Japan).

### 4.7. Monosaccharide Analysis

An ICS 5000+ ion chromatography (IC) system (Thermo Fisher Scientific, USA) was employed for monosaccharide analysis. The electrochemical detector was equipped with a gold working electrode and a pH/Ag/AgCl composite reference electrode. A CarboPac PA 10 guard column (50 mm × 4 mm) and CarboPac PA 10 (250 mm × 4 mm) separation column (Thermo Fisher Scientific) were used for sugar separation. The system was operated in the gradient mode as follows: 200 mmol·L^−1^ NaOH from −30 to −15.1 min, 18 mmol·L^−1^ NaOH from −15 to 0 min, and 18 mmol·L^−1^ NaOH from 0 to 20 min with a flow rate of 1.0 mL·min^−1^. The column and the cell of the pulsed amperometric detector were maintained at temperatures of 30°C and 35°C, respectively.

### 4.8. TMT-Based and Label-Free Quantitative Proteomic Analysis

TMT-based quantitative proteomics was conducted to study changes in the expression of proteins in *C. alburnus-*A and *C. alburnus-*B at five time points (unfertilized, 0, 0.5, and 1 h post fertilization, and ovarian tissues, *n* = 3), and label-free quantitative proteomics was conducted to study the changes in the expression of proteins in the egg envelopes of *C. alburnus-*A and *C. alburnus-*B at 1 h post fertilization. Samples in lysis buffer (8 M urea, 1% protease inhibitor cocktail) placed on ice were sonicated three times using a high-intensity ultrasonic processor (Scientz). The remaining debris was removed by centrifugation at 12000 g at 4°C for 10 min. The supernatant was collected, and the protein concentration was determined with a BCA kit according to the manufacturer's instructions. Subsequently, 100 *μ*g of protein from each sample was reduced with dithiothreitol, alkylated with iodoacetamide, digested with trypsin, and labelled with TMT reagents (Thermo, USA) (vitelline envelope samples for label-free quantitative proteomics do not require TMT labelling). The tryptic peptides were fractionated by high-pH reverse-phase HPLC using an Agilent 300Extend C18 column. The peptides were subjected to a nanospray ionization (NSI) source, followed by MS/MS in a Q Exactive™ Plus system (Thermo) coupled online to an ultraperformance liquid chromatography (UPLC) system. Proteins in fish egg and ovary with a fold change > 1.2 or < 0.83 and *p* < 0.05 and proteins in the egg envelope with a fold change > 1.5 or < 0.67 and *p* < 0.05 were considered differentially expressed (Tables S2–S4). The functions of differentially expressed proteins were determined via GO and KEGG pathway analyses and functional enrichment analysis. The GO classification was performed using the UniProt-GOA database (http://www.ebi.ac.uk/GOA/), and InterProScan software was used to predict the subcellular localization of the proteins. For quality control purposes in egg proteomics, the mass errors and lengths of all peptides were analysed. The distribution of the mass error was close to zero and mostly less than 10 ppm (Figure S9), which indicated that the mass accuracy of the MS data was sufficient. Additionally, the lengths of most peptides were distributed between 8 and 20 amino acid residues (Figure S9), which agree with the properties of tryptic peptides, indicating that the results met the standard. For quality control purposes in the proteomic analyses of egg envelopes, the mass errors were mostly less than 10 ppm (Figure S10) and the lengths of most peptides were distributed between 7 and 20 amino acid residues (Figure S10), which meant that the results met the standard.

### 4.9. RNA Extraction and Transcriptome Sequencing, Assembly, and Annotation

The total RNA (*n* = 3) of eggs and ovarian tissues was isolated using TRIzol (Invitrogen, USA) according to the manufacturer's instructions. The quantity and quality of the total RNA were verified on an Agilent 2100 Bioanalyzer and by gel electrophoresis, respectively. Approximately 10 *μ*g of each RNA sample was pooled for transcriptome library preparation. The Iso-Seq library was prepared according to the Isoform Sequencing protocol (Iso-Seq) using the Clontech SMARTer PCR cDNA Synthesis Kit and the BluePippin Size Selection System protocol, as described by Pacific Biosciences (PN 100-092-800-03). Gene functions were annotated based on the following databases: Nr (NCBI nonredundant protein sequences), Nt (NCBI nonredundant nucleotide sequences), Pfam (protein family); KOG/COG (Clusters of Orthologous Groups of proteins), Swiss-Prot (a manually annotated and reviewed protein sequence database), KO (KEGG Ortholog), and GO (Gene Ontology). For CDS prediction, the ANGEL pipeline, a long-read implementation of ANGLE, was used to determine protein coding sequences from cDNAs. We used confident protein sequences from the studied species or closely related species for ANGEL training and then ran ANGEL prediction for given sequences. For transcription factor (TF) analysis, animal TFs were analysed using the animal TFDB 2.0 database. For coding potential analysis, coding-non-coding-index (CNCI) profiles adjoining nucleotide triplets were used to effectively distinguish protein-coding and noncoding sequences independent of known annotations. We used CNCI with default parameters. The coding potential calculator (CPC) mainly assesses the extent and quality of ORFs in a transcript and searches the sequences against known protein sequence databases to clarify the coding and noncoding transcripts. We used the NCBI eukaryote protein database and set the *e*-value to 1*e* − 10 in our analysis. We translated each transcript into all three possible frames and used Pfam Scan to identify the occurrence of any of the known protein family domains documented in the Pfam database. Any transcript with a Pfam hit was excluded in the following steps. For the Pfam searches, default parameters of −*E* 0.001 to dom*E* 0.001 were used. For simple sequence repeat (SSR) analysis, SSRs in the transcriptome were identified using the MISA microsatellite finder. This tool allows the identification and localization of perfect microsatellites as well as compound microsatellites that are interrupted by a certain number of bases. For gene expression analysis, the gene expression level was normalized by using FPKM (expected number of fragments per kilobase of transcript sequence per millions base pairs sequenced) method. DESeq was used to identify differentially expressed genes with a *p* − adjusted < 0.05.

### 4.10. Preparation of Antibodies and Immunofluorescence

Two kinds of anti-ZP antibodies were synthesized in rabbits for the *C. alburnus* ZP proteins (ZP2 and ZP3X1, differentially expressed proteins in the egg envelopes from Table S3) and prepared by Dia-An Biotech Inc., Wuhan, China. Synthetic peptides designed from the amino acid sequences of the two ZP proteins were used as the antigens. Their peptide sequences were as follows: ZP2: QNFNQRTGLKTDC and YQQGRKGVPSSPPDPE; ZP3X1: SANGGGDDGDEDFRDGFV. Unfertilized eggs of *C. alburnus* were fixed in 4% paraformaldehyde in PBS at 4°C overnight, dehydrated through a graded ethanol series, and embedded in paraffin. Five-micrometer sections were placed on slides, and the samples dried. After dewaxing and rehydration, the sections were subjected to antigen retrieval. After washing in PBS, the sections were incubated in a 1 : 100 dilution of 1% normal goat serum in PBS for 1 h to block nonspecific antibody staining and were then incubated with the primary antibody (anti-ZP2) diluted 1 : 100 in 1% normal goat serum in PBS at 4°C overnight. After washing in PBS, the samples were incubated for 50 min at room temperature with a horseradish peroxidase- (HRP-) conjugated secondary antibody. Cy3-TSA diluted 1 : 2000 was added, and the samples were incubated with TSA at room temperature for 10 min. After antigen retrieval, the sections were incubated in a 1 : 100 dilution of 1% normal goat serum in PBS for 1 h to block nonspecific antibody staining and were then incubated with the primary antibody (anti-ZP3X1) diluted 1 : 100 in 1% normal goat serum in PBS at 4°C overnight. After washing in PBS, the samples were incubated for 50 min at room temperature with a fluorescein isothiocyanate- (FITC-) conjugated secondary antibody. Finally, the sections were observed under a light microscope capable of fluorescence detection and differential interference (Nikon Eclipse C1, Japan).

### 4.11. Inhibitor Tests

For these tests, solutions of 100 nM bafilomycin A_1_ (Cayman Chemical, USA), 1.5 *μ*M cathepsin L inhibitor (Cayman Chemical, USA), 5 *μ*M cyclosporine A (Cayman Chemical, USA), and 200 *μ*M DIDS (Sigma-Aldrich, Germany) were prepared in 0.05% dimethyl sulfoxide (DMSO) (BioFroxx, Germany), to inhibit the activities of V-ATPase, cathepsin L, cyclophilin D, and voltage-dependent anion-selective channel protein, respectively [63–66]. Mature female zebrafish were treated with 10 *μ*L of 0.05% DMSO or the inhibitor solution by intraperitoneal injection (*n* = 3). After 17 h, zebrafish embryos (as a semibuoyant model, remarkable hydration, and identical to other semibuoyant eggs at the biochemical level, Figure S11) were obtained by artificial insemination and then immersed in 0.05% DMSO or inhibitor solutions. Immediately thereafter, photos of eggs were taken with a stereomicroscope (MODEL C-BD230, Nikon, Japan) at 1, 5, 10, 15, 20, 25, 30, and 60 min post fertilization and the membrane diameter was measured using ImageJ (k 1.45, National Institutes of Health, USA).

### 4.12. Statistical Analyses

Statistical analyses of the data were performed by using SPSS package 25.0 (SPSS, Chicago, IL, USA). All values are presented as the mean ± standard error (SEM). The Kolmogorov-Smirnov test and Levene's test were employed to check the normality and homogeneity of variances in the data, respectively. One-way analysis of variance (ANOVA) and Tukey's multiple comparison tests were applied to determine significant differences between the results for the unfertilized and fertilized groups. Significant differences were set at *p* < 0.05 (^∗^) and *p* < 0.01 (^∗∗^).

## Data Availability

Raw peptide data are deposited in the PRIDE (accession number: PXD023609). The raw RNA-Seq data for all the libraries are available through the SRA (BioProject ID: PRJNA819171).

## Supplementary Materials

Supplementary Materials Figure S1: egg type evolution and schematic diagram of the historical distribution and dispersal of the endemic East Asian clade of Cyprinidae under the development of the Yangtze River system in response to uplift of the Qinghai-Tibetan Plateau. Figure S2: diversification dynamics and macroevolutionary patterns of endemic East Asian cyprinids. Figure S3: the differences between semibuoyant eggs and adhesive eggs at unfertilized and 0, 0.5, and 1 h post fertilization in the change of perivitelline space volume. Figure S4: the different MD changes between semibuoyant and adhesive eggs in different osmotic concentrations of 1 (deionized water), 10 (aquaculture water), 290 (phosphate buffer saline), and 930 mosmol/kg (seawater). Figure S5: the different Ca^2+^, Mg^2+^, K^+^, and Na^+^ contents between adhesive and semibuoyant eggs at unfertilized and 0, 0.5, and 1 h postfertilization. Figure S6: transcriptomic analysis. Figure S7: annotation results of transcriptomic analysis. Figure S8: the synthetic pathway of the immediate precursors of GAG. Figure S9: quality control validation of mass spectrometry data in egg proteomics. Figure S10: quality control validation of mass spectrometry data in egg envelope proteomics. Figure S11: the water, protein, T-FAA contents, and total contents of Na^+^, K^+^, Ca^2+^, and Mg^2+^ of zebrafish eggs at unfertilized and 0, 0.5, and 1 h postfertilization. Table S1: the osmolality of ion and T-FAA in adhesive and semibuoyant eggs at unfertilized and 0, 0.5, and 1 h postfertilization. Table S2: differentially expressed proteins involved in the pathways of yolk protein degradation and Ca^2+^ and Mg^2+^ active transport, the molecular structure of the egg envelope permeability transition pore, and the crosslinks of microfilament-associated proteins and adhesive-related proteins in two spawning habits of *C. alburnus* eggs at unfertilized and 0, 0.5, and 1 h postfertilization. Table S3: differentially expressed proteins involved in catalysing the early steps in GAG biosynthesis in the ovary in two spawning habits of *C. alburnus*. Table S4: differentially expressed proteins involved in the crosslinks of microfilament-associated proteins and adhesive-related proteins in two spawning habits of the egg envelope of *C. alburnus*. Table S5: summary of sequencing of *C. alburnus* transcriptome. Table S6: transcript levels for enzymes involved in biosynthesis of heparan sulphate and chondroitin/dermatan sulphates in the ovary in two spawning habits of *C. alburnus*.
