# Supplementary material for: Molecular Mechanisms of Spawning Habits for the Adaptive Radiation of Endemic East Asian Cyprinid Fishes
Source: Research (Wash D C). 2022 Sep 12;2022:9827986. doi: 10.34133/2022/9827986 (PMC9513835; doi:10.34133/2022/9827986)
Supplement: Supplementary Materials — Figure S1: egg type evolution and schematic diagram of the historical distribution and dispersal of the endemic East Asian clade of Cyprinidae under the development of the Yangtze River system in response to uplift of the Qinghai-Tibetan Plateau. Figure S2: diversification dynamics and macroevolutionary patterns of endemic East Asian cyprinids. Figure S3: the differences between semibuoyant eggs and adhesive eggs at unfertilized and 0, 0.5, and 1 h post fertilization in the change of perivitelline space volume. Figure S4: the different MD changes between semibuoyant and adhesive eggs in different osmotic concentrations of 1 (deionized water), 10 (aquaculture water), 290 (phosphate buffer saline), and 930 mosmol/kg (seawater). Figure S5: the different Ca2+, Mg2+, K+, and Na+ contents between adhesive and semibuoyant eggs at unfertilized and 0, 0.5, and 1 h postfertilization. Figure S6: transcriptomic analysis. Figure S7: annotation results of transcriptomic analysis. Figure S8: the synthetic pathway of the immediate precursors of GAG. Figure S9: quality control validation of mass spectrometry data in egg proteomics. Figure S10: quality control validation of mass spectrometry data in egg envelope proteomics. Figure S11: the water, protein, T-FAA contents, and total contents of Na+, K+, Ca2+, and Mg2+ of zebrafish eggs at unfertilized and 0, 0.5, and 1 h postfertilization. Table S1: the osmolality of ion and T-FAA in adhesive and semibuoyant eggs at unfertilized and 0, 0.5, and 1 h postfertilization. Table S2: differentially expressed proteins involved in the pathways of yolk protein degradation and Ca2+ and Mg2+ active transport, the molecular structure of the egg envelope permeability transition pore, and the crosslinks of microfilament-associated proteins and adhesive-related proteins in two spawning habits of C. alburnus eggs at unfertilized and 0, 0.5, and 1 h postfertilization. Table S3: differentially expressed proteins involved in catalysing the early steps in [file 9827986.f1.docx]

Supplementary Materials for

**Molecular Mechanisms of Spawning Habits for the Adaptive Radiation of Endemic East Asian Cyprinid Fishes**

Feng Chen, Yeke Wang, Jun He, Liang Chen, Ge Xue, Yan Zhao, Yanghui Peng, Carl Smith, Jia Zhang, Jun Chen*, Ping Xie*

*Corresponding author. Email: [xieping@ihb.ac.cn](mailto:xieping@ihb.ac.cn); chenjun@ihb.ac.cn

**This PDF file includes:**

Figures S1 to S11

Tables S1 to S6


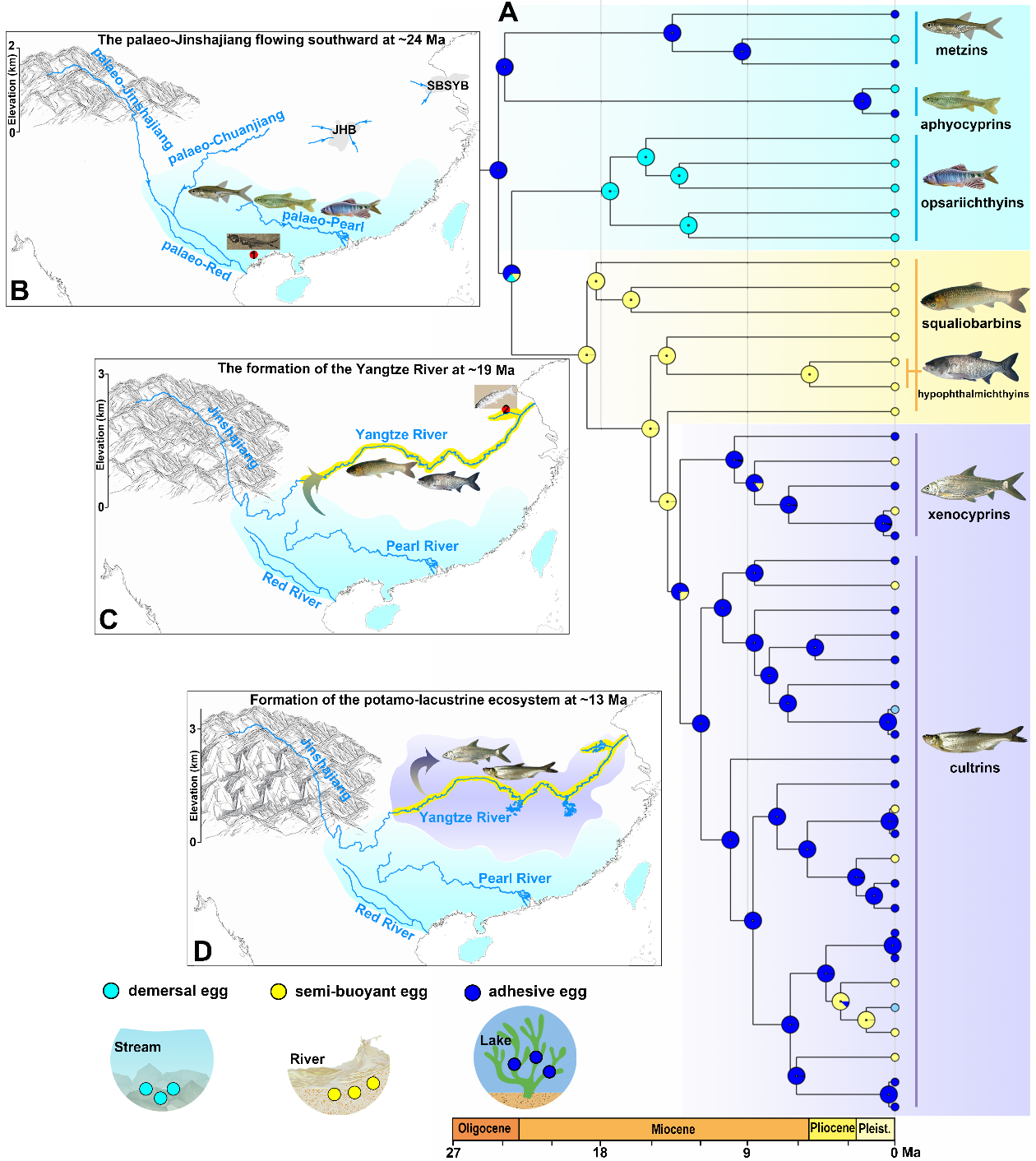


**Figure S1. Egg type evolution and schematic diagram of the historical distribution and dispersal of the endemic East Asian clade of Cyprinidae under the development of the Yangtze River system in response to uplift of the Qinghai-Tibetan Plateau.** (**A**) Egg type evolution of the endemic East Asian cyprinids. (**B-D**) Schematic diagram of the historical distribution and dispersal of endemic East Asian cyprinids during the development of the Yangtze River system during uplift of the Qinghai-Tibetan Plateau. Different colored regions represent different groups (A) and their ancestral distribution (B-D). Red dots indicate fossil sites of endemic East Asian cyprinid fish, including the Ningming Basin, Guangxi Province (B, 1) and Sihong Basin, Jiangsu Province (C, 2). JHB, Jianghan Basin; SBSYB, Subei-South Yellow Sea Basin (B). These results originate from doi: 10.21203/rs.3.rs-145035/v3.


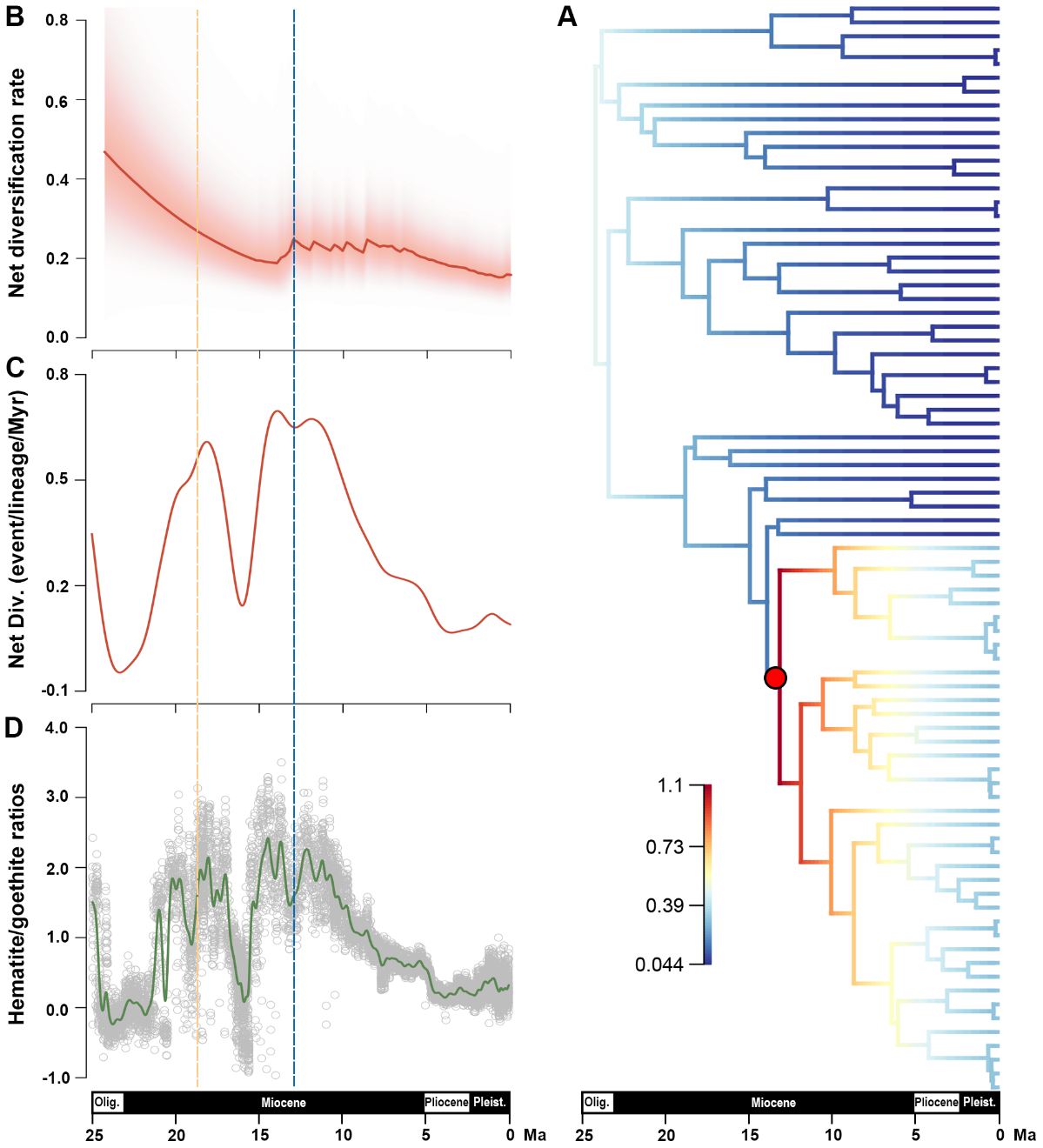


**Figure S2. Diversification dynamics and macroevolutionary patterns of endemic East Asian cyprinids.** (A) A single rate shift configuration with the maximum a posteriori probability represented as a phylorate plot of the endemic East Asian cyprinids showing variation in speciation rates. Warmer colors represent higher rates. A red dot denotes the estimated position of the single rate shift configuration that has the highest posterior probability in the 95% credible set of shift configurations. (B) The rate-through-time plot of the endemic East Asian cyprinids implemented by BAMM. (C) Net diversification rate through time for the endemic East Asian cyprinids inferred from the relationship between the net diversification rate and East Asian monsoon in *RPANDA*. (D) The hematite/goethite proxy of ODP Site 1148 in the South China Sea is positively correlated with the intensity of the East Asian monsoons as a function of time. The yellow dotted-line indicates the appearance of the semi-buoyant egg group at ~19 Ma. The blue dotted-line represents the time that fishes with adhesive eggs appeared again, and the net diversification rate of the endemic East Asian cyprinids peaked at ~13 Ma. These results originate from doi: 10.21203/rs.3.rs-145035/v3.

**
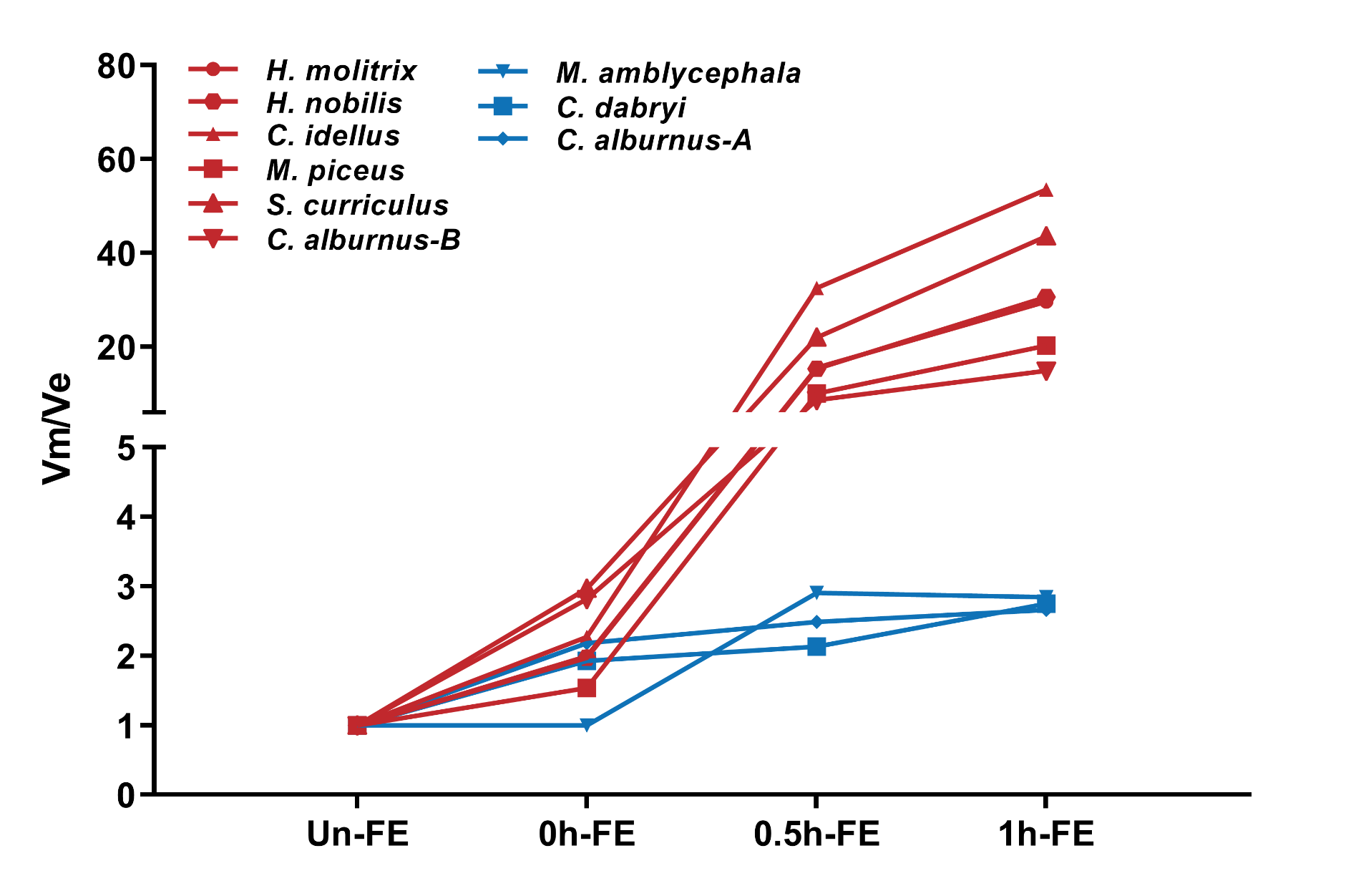
**

**Figure S3.** **The differences between semi-buoyant eggs (red lines) and adhesive eggs (blue lines) at unfertilized (Un-FE), and 0, 0.5 and 1 h postfertilization (0 h-FE, 0.5 h-FE, and 1 h-FE) in the change of perivitelline space volume.** $\boldsymbol{V=}\frac{\boldsymbol{4}}{\boldsymbol{3}}\boldsymbol{\pi}\boldsymbol{r}^{\boldsymbol{3}}$, the radiuses of Vm and Ve are MD/2 and ED/2, respectively. Values are means ± SEMs from two to three separate experiments (5-8 eggs per group in each experiment).

**
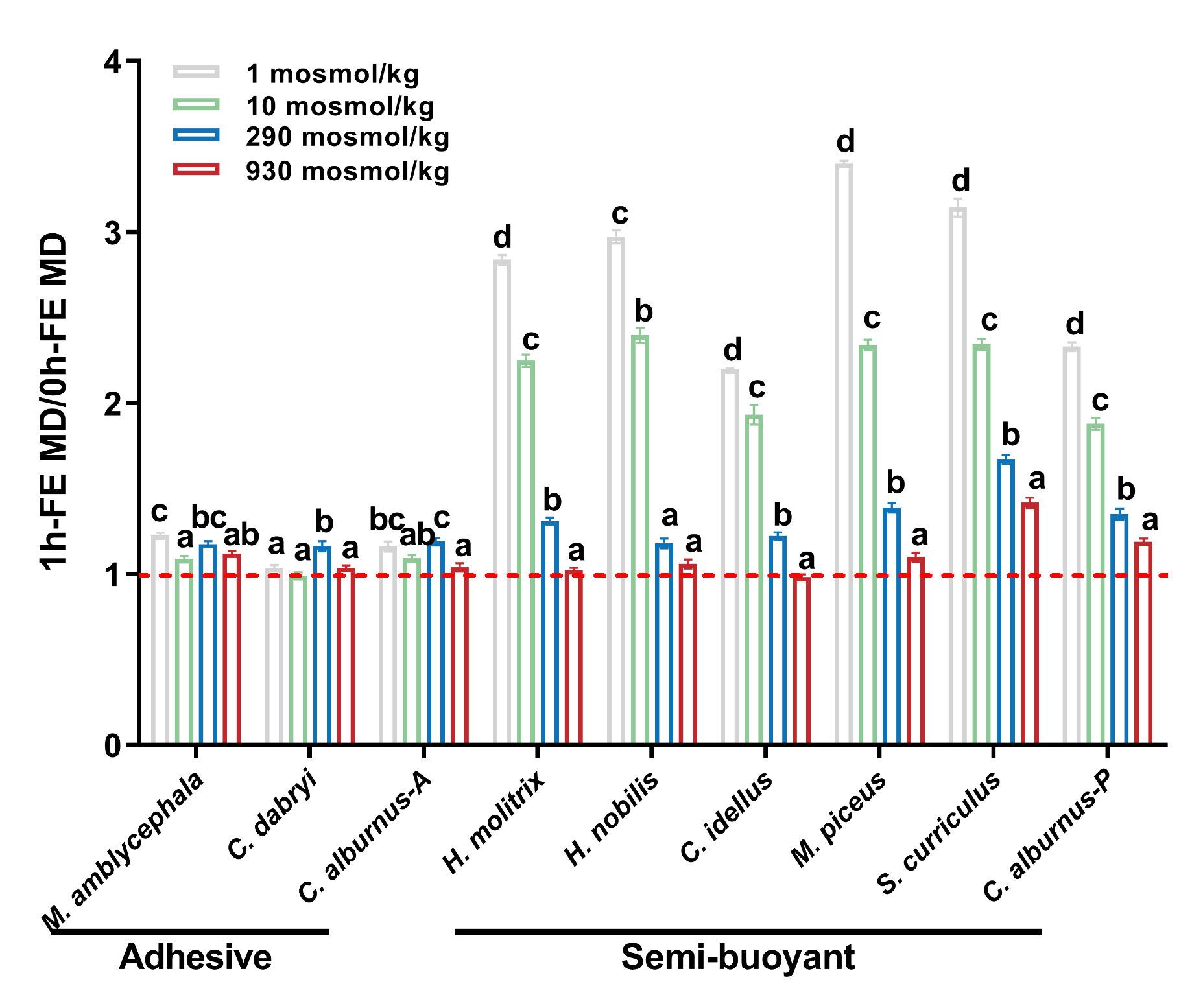
**

**Figure S4. The different MD changes between semi-buoyant and adhesive eggs in different osmotic concentrations of 1 (deionized water), 10 (aquaculture water), 290 (phosphate buffer saline), 930 mosmol/kg (seawater).** Values are means ± SEMs (n = 10 eggs). Different lower-case letters indicate significant differences between different osmotic concentrations (*p* < 0.05, one-way analysis of variance). The horizontal dashed red line indicates the MDs of eggs 0.5 h and 1 h after fertilization were identical.


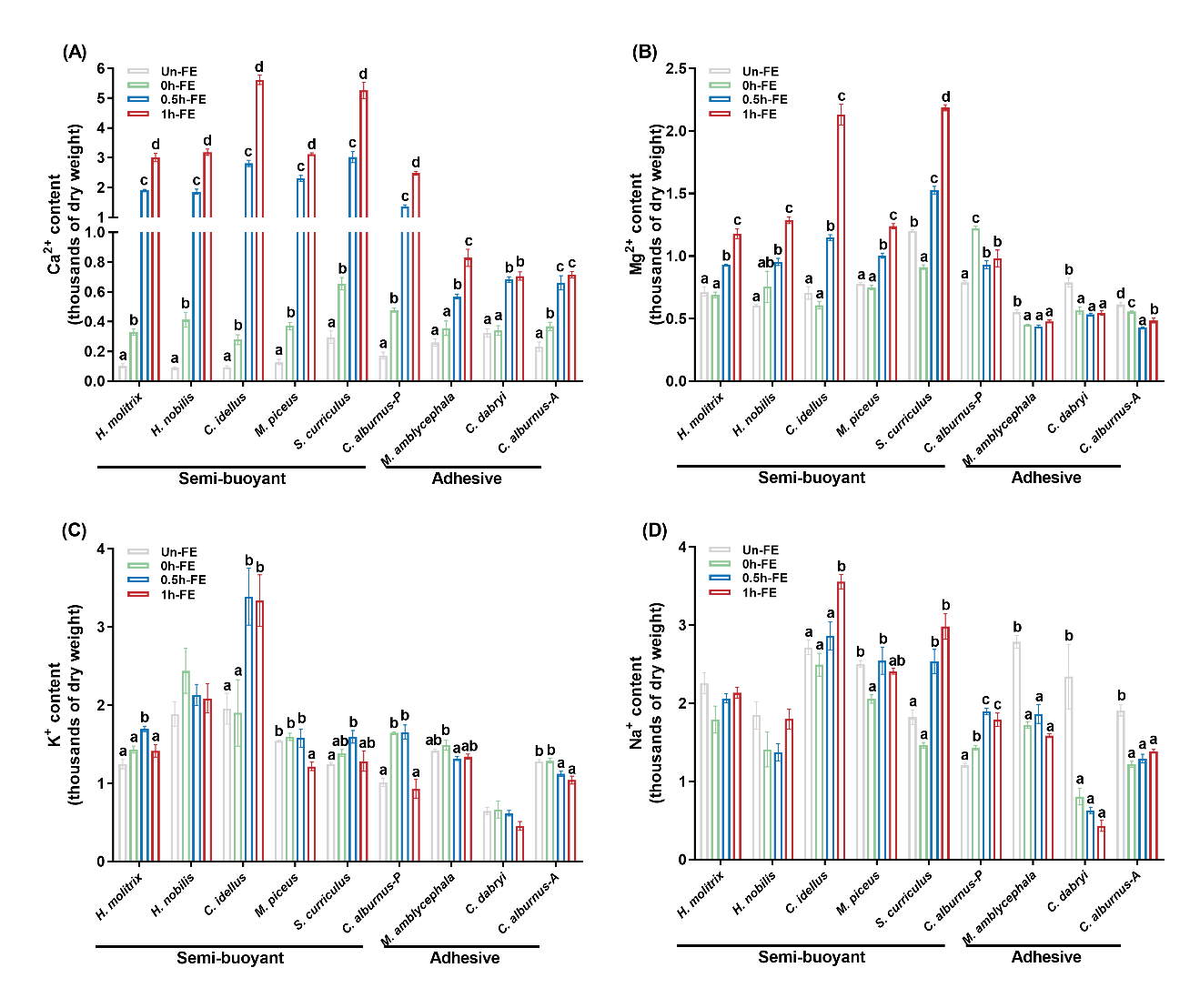


**Figure S5. The different Ca^2+^, Mg^2+^, K^+^ and Na^+^ contents between adhesive and semi-buoyant eggs at unfertilized (Un-FE), and 0, 0.5 and 1 h postfertilization (0 h-FE, 0.5 h-FE, and 1 h-FE).** (A) Ca^2+^ content. (B) Mg^2+^ content. (C) K^+^ content. (D) Na^+^ content. Values are means ± SEMs from four separate experiments. Different lower-case letters indicate significant differences between the four stages (*p* < 0.05, one-way analysis of variance).


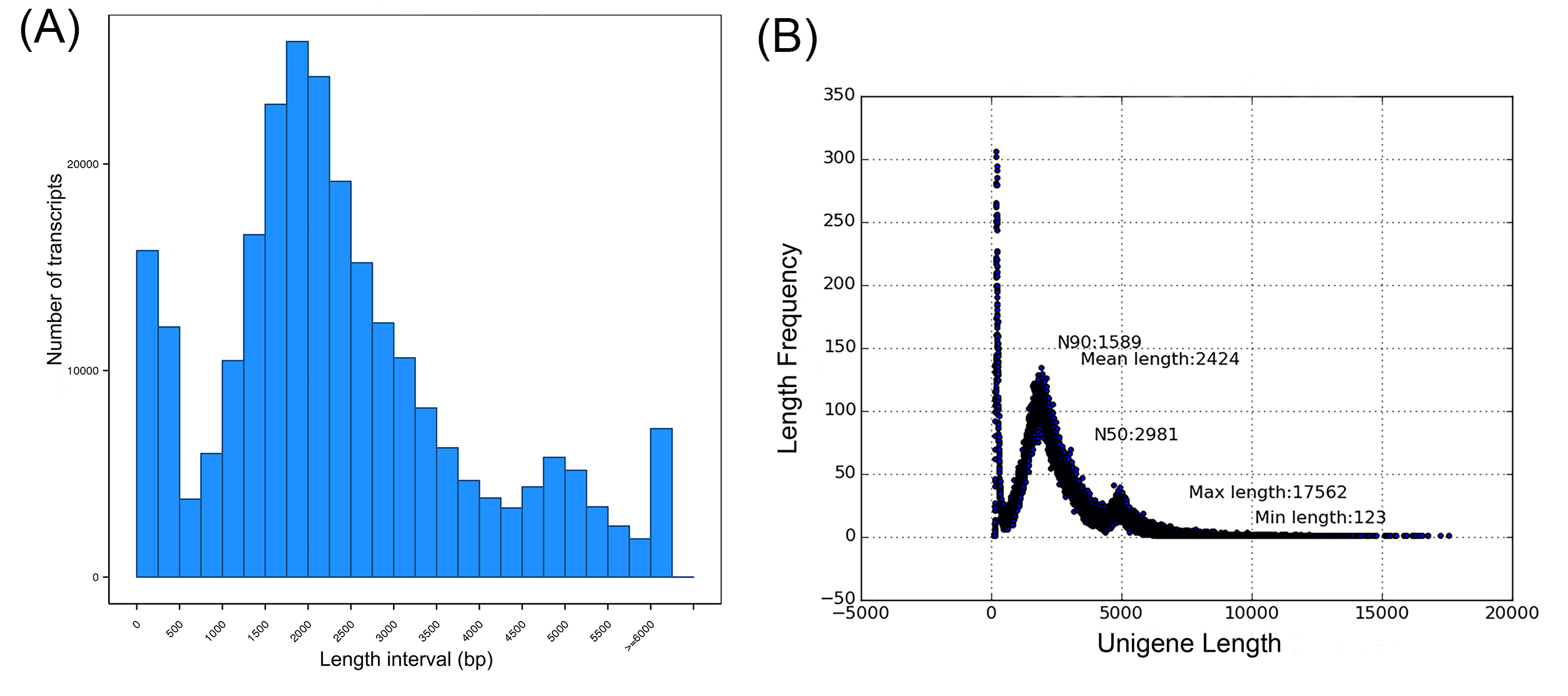


**Figure S6.** **Transcriptomic analysis.** (A) Length distribution of all transcripts for *C. alburnus* and (B) cumulative length of unigenes for *C. alburnus*.


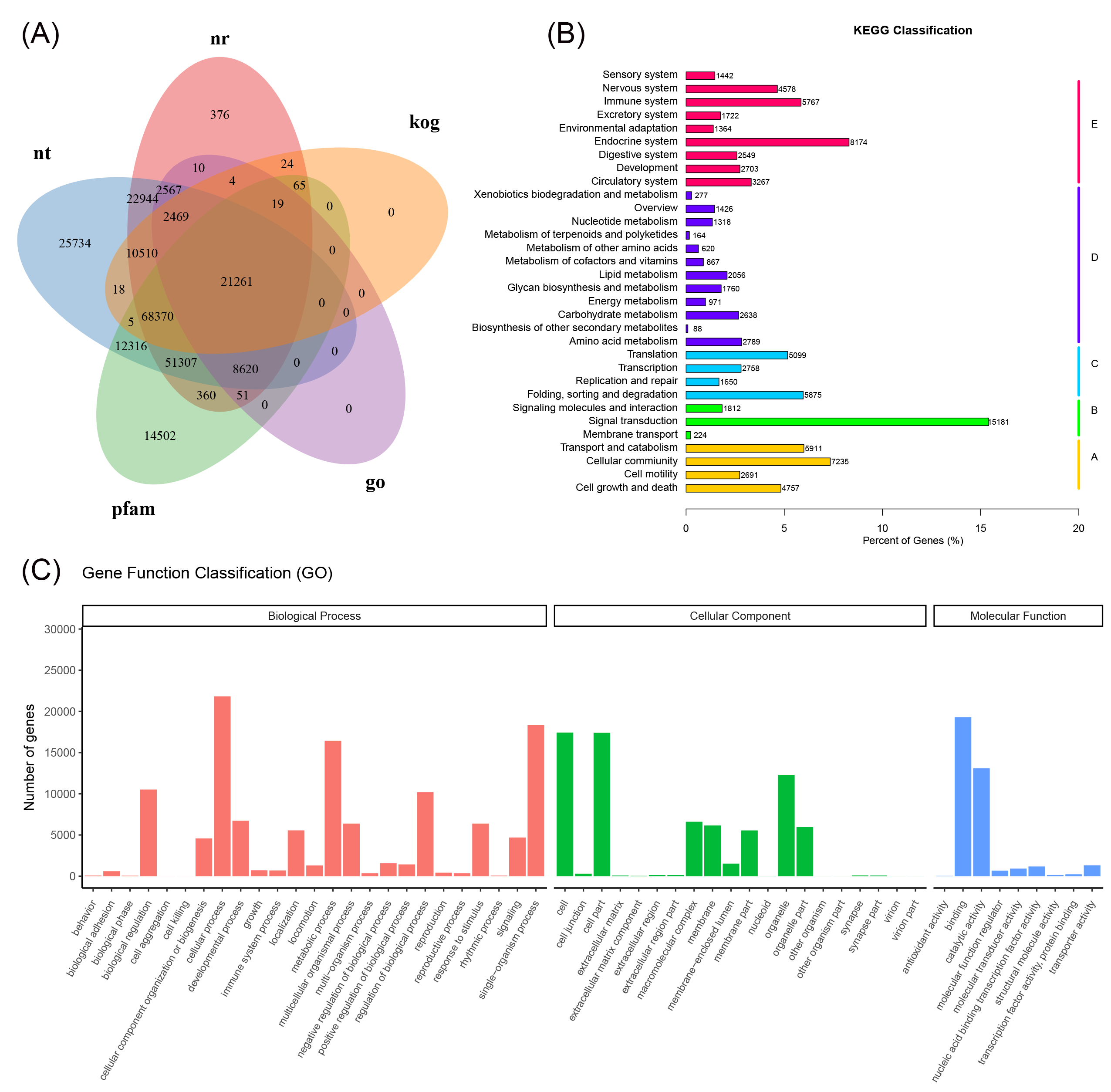


**Figure S7.** **Annotation results of transcriptomic analysis.** (A) Venn diagram drawn from 5 databases, including Nr, Nt, Pfam, KOG/COG and GO. (B) KEGG classification of assembled unigenes in the *C. alburnus* transcriptome. (C) GO classification of assembled unigenes in the *C. alburnus* transcriptome.


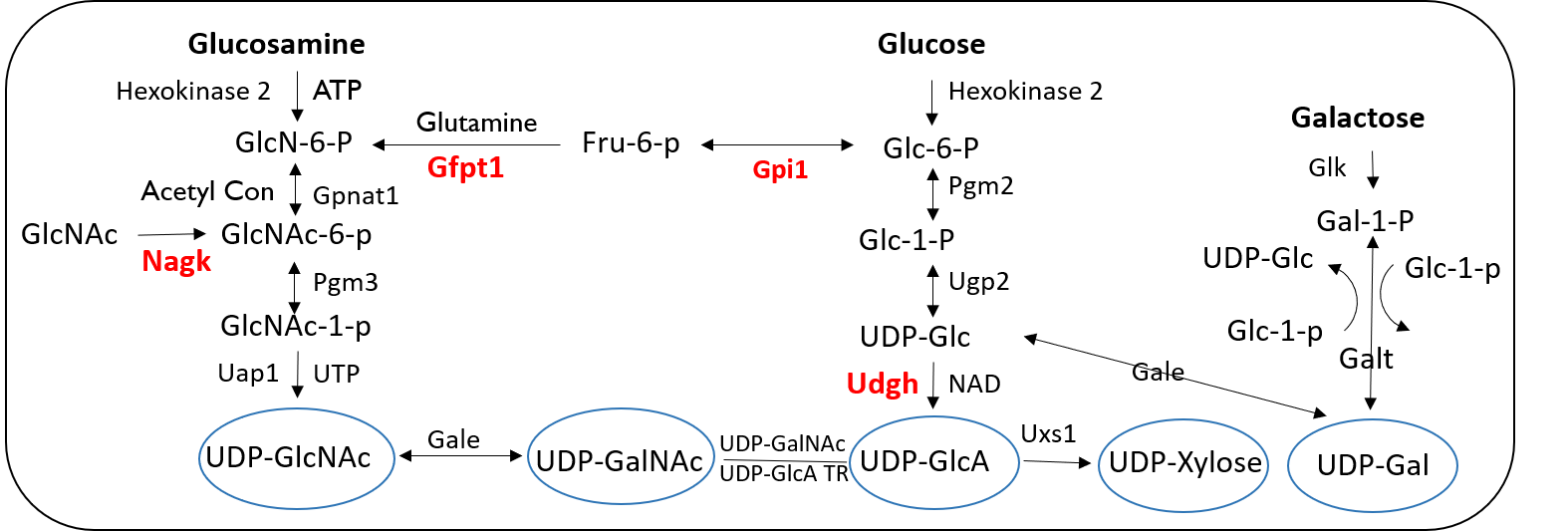


**Figure S8. The synthetic pathway of the immediate precursors of GAG. Five precursors of GAG biosynthesis are shown in ovals (UDP-GlcNAc, UDP-GalNAc, UDP-GlcA, UDP-Xyl, and UDP-Gal).** Red text represents up-regulated enzymes. Gfpt, glutamine--fructose-6-phosphate aminotransferase; Gpi1, glucose-6-phosphate isomerase; Nagk, N-acetylmannosamine kinase; Udgh, UDP-glucose 6-dehydrogenase; Galt, galactose-1-phosphate uridylyltransferase.


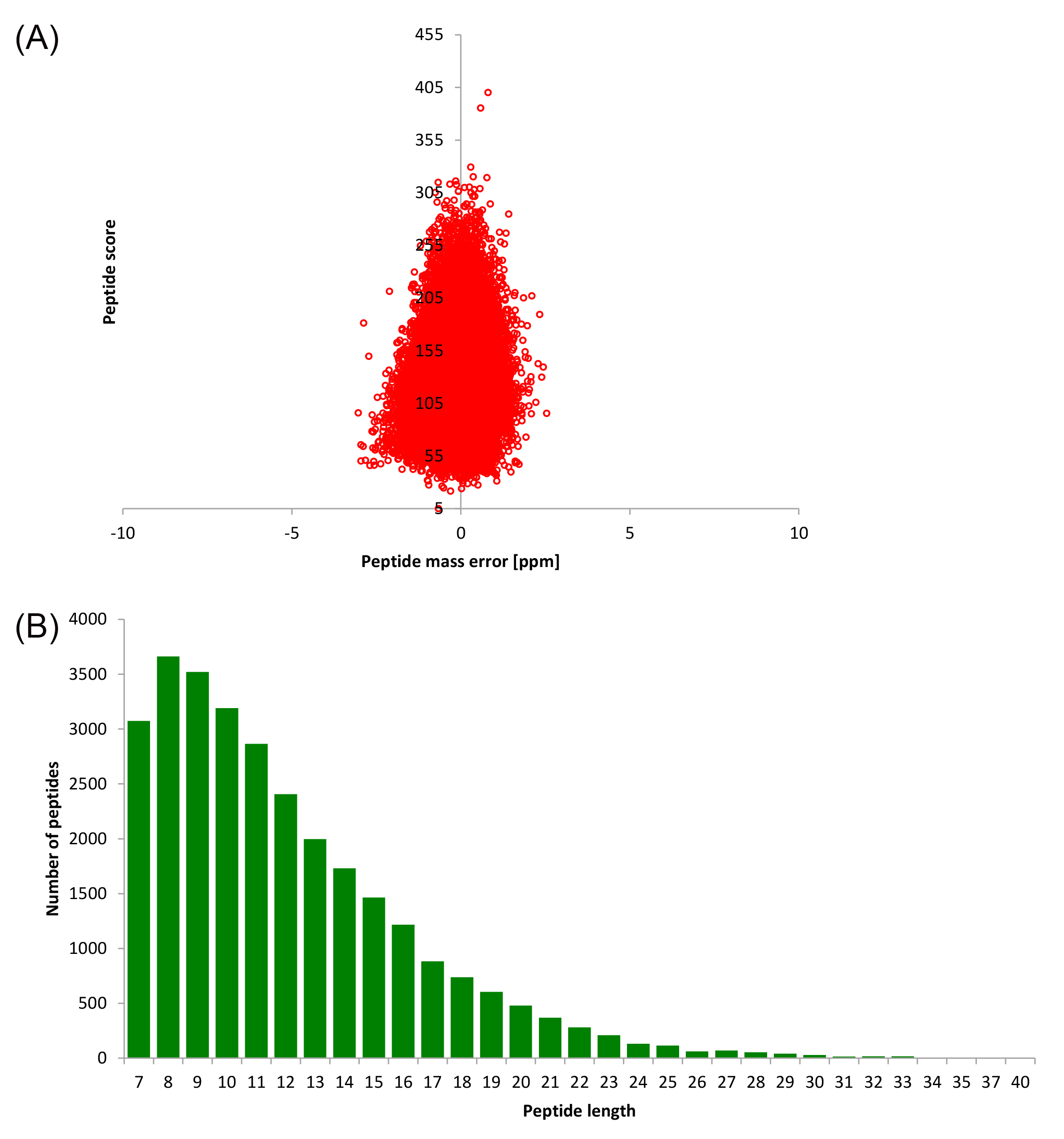


**Figure S9. Quality control validation of mass spectrometry data in egg proteomics.** (A) mass error distribution and (B) peptide lengths of peptides identified by TMT.


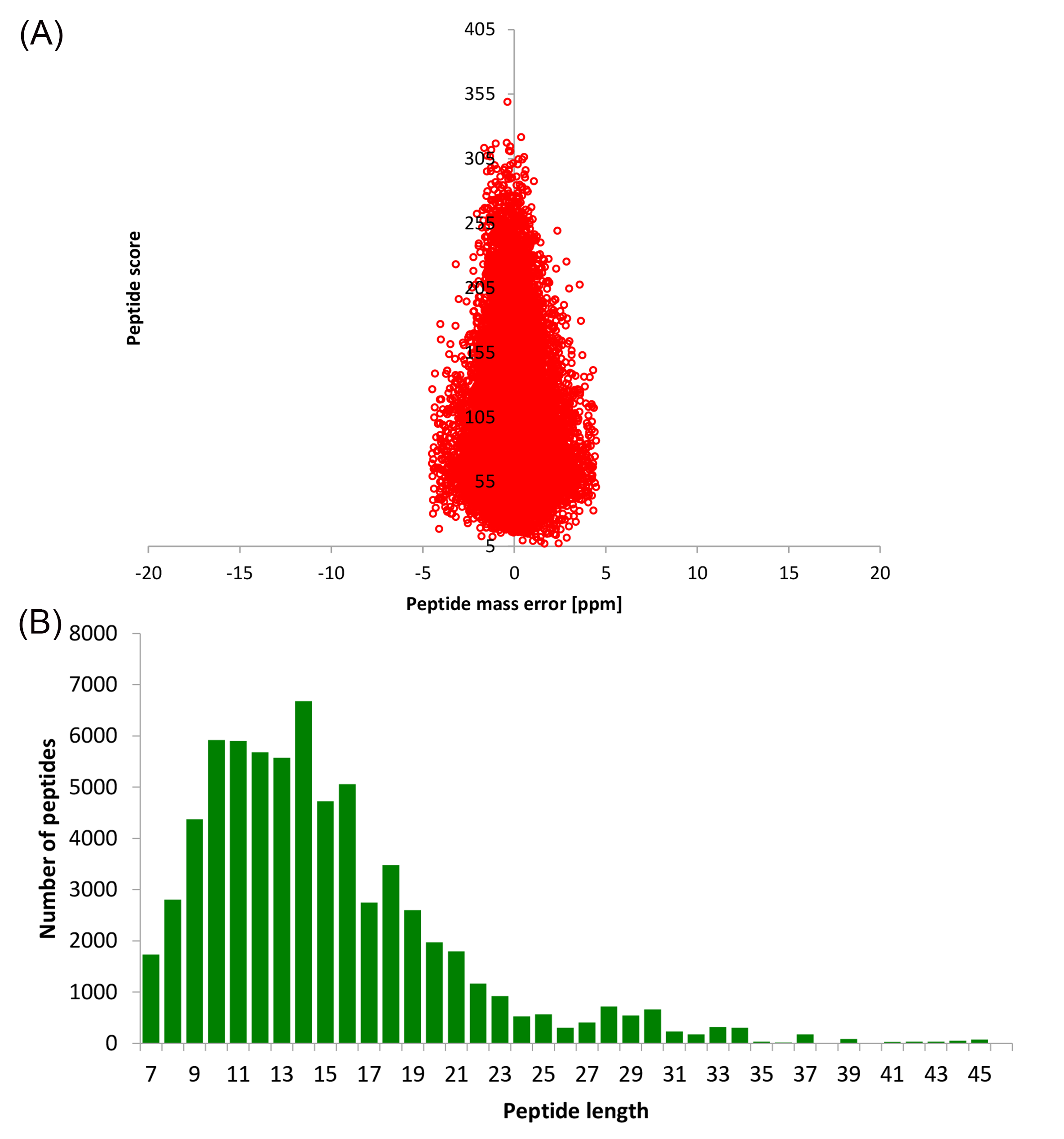


**Figure S10. Quality control validation of mass spectrometry data in egg envelope proteomics.** (A) mass error distribution and (B) peptide lengths of peptides identified by label-free.

**
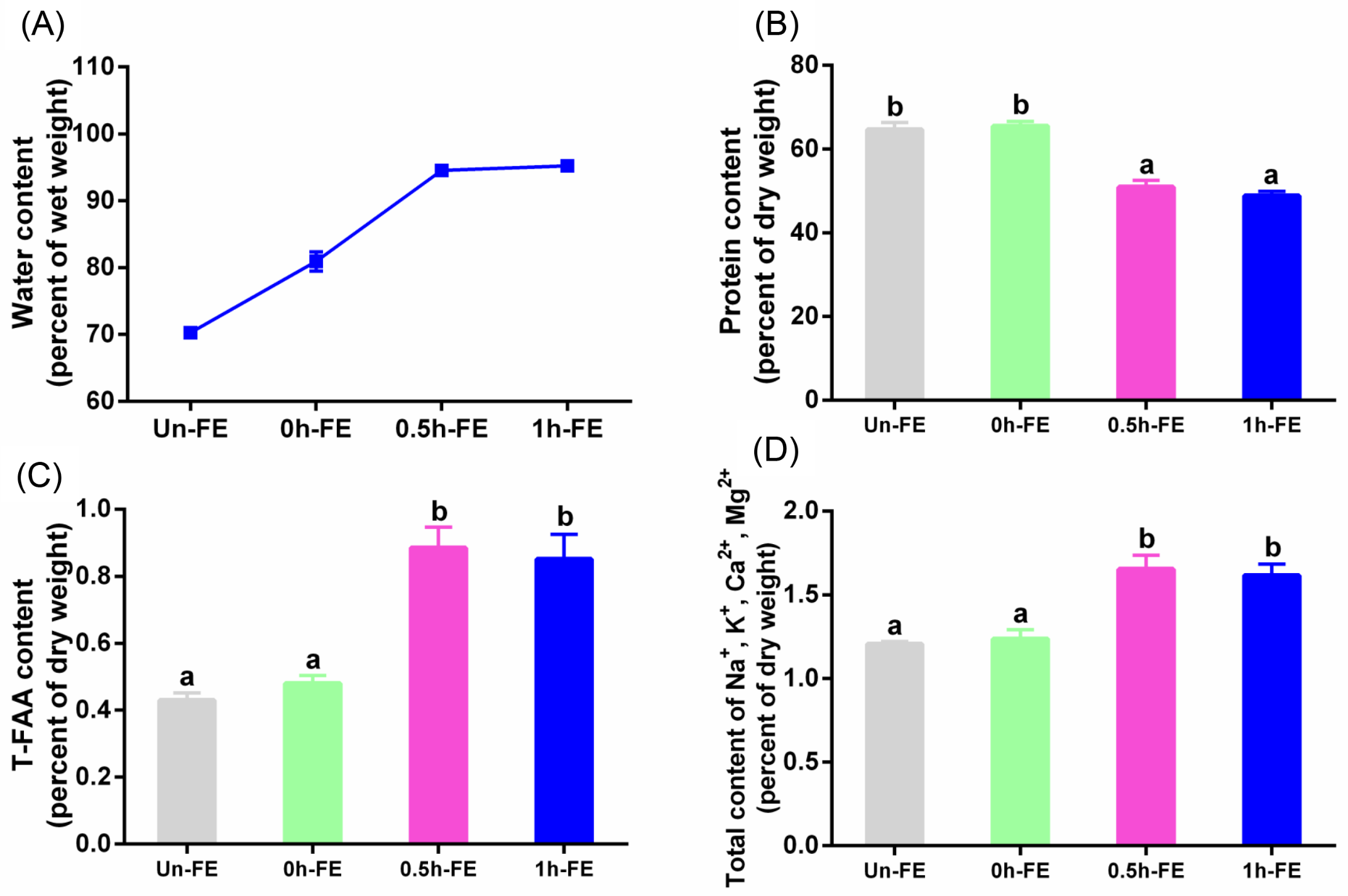
**

**Figure S11. The water (A), protein (B), T-FAA content and (C) total content of Na^+^, K^+^, Ca^2+^, and Mg^2+^ (D) of zebrafish eggs at unfertilized (Un-FE), and 0, 0.5 and 1 h postfertilization (0 h-FE, 0.5 h-FE, 1 h-FE).** Values are means ± SEMs from three to five separate experiments. Different lower-case letters indicate significant differences between four stages (*p* < 0.05, one-way analysis of variance). T-FAA, total free amino acids.

**Table S1. The osmolality (means ± SD, mosmol/kg) of ion and T-FAA in adhesive and semi-buoyant eggs at unfertilized, and 0, 0.5 and 1 h postfertilization.**

|  | **Un-FE** | |  | **0h-FE** | |  | **0.5h-FE** | |  | **1h-FE** | |
| --- | --- | --- | --- | --- | --- | --- | --- | --- | --- | --- | --- |
| **Species** | **Ion** | **T-FAA** |  | **Ion** | **T-FAA** |  | **Ion** | **T-FAA** |  | **Ion** | **T-FAA** |
| **Adhesive** |  |  |  |  |  |  |  |  |  |  |  |
| *M. amblycephala* | 101.28±0.66 | 15.05±0.31** |  | 66.86±1.12 | 14.25±1.98** |  | 48.29±3.15 | 9.01±0.81** |  | 41.77±1.46 | 8.54±0.69** |
| *C. dabryi* | 70.18±14.17 | 11.12±0.44** |  | 38.77±5.39 | 12.40±0.23** |  | 23.05±0.97 | 7.60±0.43** |  | 17.29±1.64 | 7.56±1.07** |
| *C. alburnus-A* | 84.91±2.84 | 14.59±0.37** |  | 57.54±1.13 | 10.60±0.80** |  | 38.23±1.40 | 6.83±0.29** |  | 29.82±1.57 | 5.67±0.41** |
| **Semi-buoyant** |  |  |  |  |  |  |  |  |  |  |  |
| *H. molitrix* | 96.81±5.17 | 20.51±0.84** |  | 75.63±5.04 | 19.53±0.89** |  | 21.00±0.38 | 10.35±1.22** |  | 13.94±0.24 | 5.64±0.12** |
| *H. nobilis* | 86.31±1.07 | 14.94±1.61** |  | 46.19±2.29 | 14.48±0.27** |  | 17.55±0.81 | 3.97±0.12** |  | 14.04±0.94 | 2.46±0.03** |
| *C. idellus* | 99.51±6.79 | 17.45±3.94** |  | 76.81±9.28 | 17.01±5.00** |  | 15.00±0.69 | 2.68±0.28** |  | 11.05±0.87 | 2.14±0.08** |
| *M. piceus* | 112.11±1.61 | 14.35±0.46** |  | 84.39±2.95 | 18.58±1.38** |  | 20.77±1.80 | 3.43±0.32** |  | 14.20±0.22 | 2.48±0.17** |
| *S. curriculus* | 100.48±3.31 | 21.42±1.90** |  | 54.91±1.28 | 18.39±0.63** |  | 16.88±0.98 | 3.30±0.11** |  | 11.98±0.39 | 1.95±0.20** |
| *C. alburnus-B* | 70.94±2.30 | 15.31±0.46** |  | 52.02±1.01 | 15.10±0.55** |  | 17.95±0.50 | 5.46±0.10** |  | 12.43±0.85 | 3.67±0.15** |

Values are calculated by solute concentrations from three to four separate experiments. Ion content is double that of cations (Na^+^, K^+^, Ca^2+^, Mg^2+^) and an osmotic coefficient of 0.9 for monovalent ions. Asterisks indicate significant differences between the osmolality of ions and T-FAA (** *p* < 0.01, T-statistics).

**Table S2. Expressed proteins in two spawning habits of *C. alburnus* eggs at unfertilized (Un-FE), and 0, 0.5 and 1 h postfertilization (0 h-FE, 0.5 h-FE, and 1 h-FE).**

| **Protein accession** | **Protein description** | **Abbreviation** | **Fold change^a^** | | | |
| --- | --- | --- | --- | --- | --- | --- |
|  |  |  | BE-Un/  AE-Un | BE-0h/  AE-0h | BE-0.5h/  AE-0.5h | BE-1h/  AE-1h |
| **The pathways of yolk protein degradation** | | | | | | |
| **Vtg degradation pathway** | | | | | | |
| AE_0_Cluster-211.16816 | vitellogenin | Vtg | 0.80** | 1 | 0.97 | 0.88 |
| AE_60_Cluster-18666.0 | vitellogenin B1 | VtgB1 | 0.81** | 0.76** | 0.72** | 0.83** |
| AE_60_Cluster-11526.1667 | vitellogenin receptor | Vtgr | 1.56** | 1.63** | 1.69** | 1.47** |
| BE_O_Cluster-7693.2710 | clathrin heavy chain 1-like | CHC1-l | 1.32 | 1.34* | 1.23 | 1.27** |
| BE_Unf_Cluster-8245.10580 | clathrin light chain A isoform X2 | CLCAX2 | 1.19 | 1.18 | 1.30** | 1.19 |
| BE_Unf_Cluster-8245.12396 | clathrin light chain B isoform X2 | CLCBX2 | 0.74 | 1.09 | 1.73 | 1.38* |
| AE_O_Cluster-4339.18256 | low-density lipoprotein receptor-like | LDLR-l | 1.85 | 2.31** | 1.97** | 1.05 |
| AE_O_Cluster-4339.9396 | low-density lipoprotein receptor-related protein 2 isoform X2 | LDLRP2X2 | 2.11** | 2.19** | 2.02** | 2.00** |
| BE_60_Cluster-3379.16529 | V-type proton ATPase subunit C 1-A-like | V-ATPase C1A-l | 0.83 | 0.96 | 0.93 | 1.50** |
| AE_O_Cluster-4339.24312 | V-type proton ATPase subunit E 1-like | V-ATPase E1-l | 1.23** | 1.22 | 1.32** | 1.28 |
| BE_Unf_Cluster-11065.0 | L-cystatin-like | CstL-l | 0.82 | 0.62** | 1.06 | 0.77* |
| BE_Unf_Cluster-9981.0 | cystatin C precursor | CstC-p | 0.98 | 0.87 | 0.53* | 0.60** |
| BE_60_Cluster-3379.14304 | cathepsin K | CatK | 0.81 | 1.46 | 1.33 | 1.87** |
| BE_Unf_Cluster-8245.9418 | cathepsin L-1 like | CatL-1l | 1.32* | 1.81** | 1.47** | 2.14** |
| BE_O_Cluster-7693.23861 | cathepsin S-like | CatS-l | 1.08 | 1.39* | 0.99 | 1.2 |
| **zinc metalloproteinase pathway** | | | | | | |
| AE_O_Cluster-4339.9887 | zinc finger protein 622 | ZFP622 | 1.43* | 1.30* | 1.14 | 1.27* |
| AE_Unf_Cluster-5694.5914 | zinc finger protein ZPR1 | ZFP-ZPR1 | 1.32 | 1.16 | 1.66* | 1.34 |
| BE_30_Cluster-692.0 | zinc finger protein 638-like isoform X2 | ZFP638-lX1 | 1.31 | 0.72 | 1.46* | 0.99 |
| AE_30_Cluster-7470.13825 | alpha-2-macroglobulin-like | α2M-l | 0.13** | 0.11** | 0.09** | 0.14** |
| AE_60_Cluster-11526.9075 | alpha-2-macroglobulin-like protein 1 isoform X1 | α2M-l1X1 | 0.10** | 0.09** | 0.09** | 0.07** |
| AE_0_Cluster-211.4023 | alpha-2-macroglobulin-like precursor | α2M-lp | 0.13** | 0.06** | 0.08** | 0.15* |
| AE_O_Cluster-4339.17835 | alpha-2-macroglobulin-like, partial | α2M-l | 0.18** | 0.14** | 0.13** | 0.24** |
| BE_Unf_Cluster-8245.1077 | metalloproteinase inhibitor 2-like | MPI2-l | 0.32** | 0.57 | 0.63 | 0.3 |
| BE_60_Cluster-3379.9219 | zinc metalloproteinase nas-14-like isoform X1 | ZMP14-lX1 | 6.38* | 5.72* | 4.98* | 4.26** |
| **ubiquitin-proteasome pathway** | | | | | | |
| BE_Unf_Cluster-8245.4156 | Ubiquitin-like 1-activating enzyme E1A | E1A | 1.22** | 1.17 | 1.13 | 1.05 |
| BE_Unf_Cluster-8245.9142 | ubiquitin-conjugating enzyme E2 C | E2 C | 1.14 | 1.28** | 1.27 | 1.37* |
| BE_30_Cluster-8188.940 | ubiquitin-conjugating enzyme E2 N | E2 N | 1.31* | 0.94 | 1.30* | 1.31** |
| AE_Unf_Cluster-5694.11885 | ubiquitin-conjugating enzyme E2 T | E2 T | 0.81* | 0.75* | 0.94 | 1.07 |
| BE_Unf_Cluster-8245.5181 | ubiquitin-conjugating enzyme E2 variant 1 isoform X2 | E2 1X2 | 1.23* | 0.99 | 1.06 | 1.17 |
| AE_30_Cluster-15482.0 | ubiquitin-like-conjugating enzyme ATG3-like | E2 ATG3-l | 1.21 | 1.11 | 1.35* | 1.18 |
| AE_Unf_Cluster-5694.7278 | E3 ubiquitin-protein ligase RFWD2-like | E3 RFWD2-l | 1.34 | 1.57 | 1.12 | 1.55* |
| BE_O_Cluster-7693.17555 | E3 ubiquitin-protein ligase TRIM36 | E3 TRIM36 | 1.35** | 1.21* | 1.21 | 1.27 |
| AE_O_Cluster-4339.24137 | E3 ubiquitin-protein ligase TRIP12 isoform X1 | E3 TRIP12X1 | 1.2 | 1.37* | 1.11 | 1.03 |
| AE_Unf_Cluster-5694.6364 | ubiquitin carboxyl-terminal hydrolase 48 | UCH48 | 1.17 | 0.99 | 0.69** | 0.67 |
| BE_Unf_Cluster-8245.8191 | ubiquitin carboxyl-terminal hydrolase 7 isoform X3 | UCH7X3 | 0.99 | 0.94 | 0.88 | 0.82** |
| BE_Unf_Cluster-8245.14717 | ubiquitin carboxyl-terminal hydrolase isozyme L3 | UCHL3 | 0.95 | 0.92 | 0.81* | 0.74** |
| BE_Unf_Cluster-8245.9114 | proteasome activator complex subunit 3 | PMSE3 | 1.45* | 1.32* | 1.50** | 1.64** |
| BE_Unf_Cluster-8245.13961 | 26S proteasome non-ATPase regulatory subunit 1-like | PSMD1-l | 1.22 | 1.55* | 1.29 | 1.69** |
| BE_30_Cluster-8188.14141 | 26S proteasome non-ATPase regulatory subunit 2 | PSMD2 | 0.9 | 0.94 | 1.17 | 1.51** |
| The pathways of Ca^2+^ and Mg^2+^ active transport | | | | | | |
| AE_60_Cluster-11526.8396 | magnesium transporter protein 1 | MTP1 | 1.04 | 1.53** | 1.38* | 1.05 |
| BE_Unf_Cluster-8245.11173 | calmodulin-like | CaM-l | 0.8 | 0.97 | 0.72 | 0.63** |
| BE_O_Cluster-7693.26291 | plasma membrane calcium ATPase 4 | PMCA4 | 1.39 | 0.92 | 0.16* | 0.10** |
| BE_0_Cluster-19419.3374 | metallothionein | MT | 0.59* | 0.43** | 0.58** | 0.58** |
| **The molecular structure of** **the egg envelope permeability transition pore** | | | | | | |
| BE_60_Cluster-3379.17584 | voltage-dependent anion-selective channel protein 2-like | VDAC2-l | 0.76* | 1.14 | 1.09 | 0.86 |
| BE_Unf_Cluster-8245.1536 | voltage-dependent anion channel | VDAC | 0.78* | 1.17 | 1.15 | 0.91 |
| BE_Unf_Cluster-8245.8792 | Voltage-dependent anion-selective channel protein 1 | VDAC1 | 0.74 | 0.99 | 1.02 | 0.64* |
| BE_30_Cluster-8188.7392 | voltage-dependent anion-selective channel protein 3 | VDAC3 | 1.12 | 0.81 | 0.18 | 0.11** |
| BE_Unf_Cluster-8245.6229 | cyclophilin D | CypD | 0.94 | 0.78* | 1.02 | 0.94 |
| BE_0_Cluster-23403.0 | adenine nucleotide transporter | ANT | 0.81 | 1.09 | 1.01 | 0.73 |
| AE_O_Cluster-4339.17372 | phosphate carrier protein | PiC | 0.50** | 0.54* | 0.57* | 0.60** |
| **The** **crosslinks of microfilament-associated proteins and adhesive-related proteins** | | | | | | |
| AE_30_Cluster-7470.11324 | fibronectin | Fn-l | 0.81* | 0.77 | 1.25 | 1.18 |
| AE_O_Cluster-4339.23328 | laminin subunit beta-1 precursor | Lamβ1 | 0.76** | 0.83 | 1.39** | 1.56* |
| BE_O_Cluster-7693.28141 | neural Wiskott-Aldrich syndrome protein | WASP | 1.14 | 1.03 | 1.1 | 1.12 |
| BE_Unf_Cluster-8245.5589 | integrin alpha-5 precursor | Itgα5-l | 0.93 | 1.01 | 0.81 | 0.44 |
| AE_60_Cluster-16480.2 | integrin beta-6-like | Itgβ6-l | 1.22 | 1.25 | 1.89 | 1.92** |
| BE_O_Cluster-7693.36314 | protein-glutamine gamma-glutamyltransferase K-like | TGase-Kl | 0.68* | 1.16 | 1.45 | 1.33 |
| AE_O_Cluster-4339.10520 | collagen alpha-2(VI) chain isoform X1 | colα2VI | 0.83 | 1.15 | 2.08** | 2.13** |
| BE_O_Cluster-5905.1 | collagen alpha-3(VI) chain-like | colα3VI | 1.1 | 1.04 | 1.69* | 1.75** |
| BE_O_Cluster-7693.30229 | collagen type I alpha 2 | colα2I | 0.92 | 0.93 | 1.69* | 2.78* |

Note: ^a^ the fold changes are indicated as compared with adhesive eggs (AE). Values >1 indicate up-regulation, and values <1 indicate down-regulation. Proteins with fold difference >1.2 or <0.83 were considered significantly altered, which are indicated with * (*p* < 0.05) or ** (*p* < 0.01).

**Table S3. Differentially expressed proteins in ovary in two ecotypes of *C. alburnus*.**

| **Protein accession** | **Protein description** | **Abbreviation** | **Fold change^a^**  **AE-O /BE-O** |
| --- | --- | --- | --- |
| AE_O_Cluster-4339.28871 | glutamine--fructose-6-phosphate aminotransferase | Gfpt | 1.45** |
| AE_0_Cluster-211.11147 | Glucose-6-phosphate isomerase | Gpi1 | 5.44** |
| BE_Unf_Cluster-8245.6191 | N-acetylmannosamine kinase | Nagk | 1.32** |
| BE_Unf_Cluster-8245.4155 | UDP-glucose 6-dehydrogenase | Udgh | 1.91** |

Note: ^a^ the fold changes are indicated as compared with the *C. alburnus*-B ovary (BE-O). Values >1 indicate up-regulation, and values <1 indicate down-regulation. Proteins with fold difference >1.2 or <0.83 were considered significantly altered, which are indicated with ** (*p* < 0.01).

**Table S4.** **Differentially expressed proteins in two spawning habits of the egg envelope of** ***C. alburnus*.**

| **Protein accession** | **Protein description** | **Abbreviation** | **Fold change^a^  BE-1h-FE/AE-1h-FE** |
| --- | --- | --- | --- |
| BE_30_Cluster-8188.16556 | ARP2 actin-related protein 2 homolog | ARP2 | 0.32** |
| BE_60_Cluster-3379.9596 | alpha-actinin-4 isoform X2 | α−actn4 | 0.3** |
| AE_O_Cluster-4339.19182 | filamin-A isoform X2 | flnA | 0.41** |
| AE_O_Cluster-4339.19228 | actin, cytoplasmic 1 | Actb | 0.47** |
| BE_Unf_Cluster-8245.13635 | profilin-2 isoform X1 | pfn2X1 | 0.34** |
| BE_Unf_Cluster-8245.1931 | cofilin-2-like | cof2-l | 0.35** |
| BE_Unf_Cluster-8245.5917 | Capping protein (actin filament) muscle Z-line, alpha 1 | capz-α1 | 0.27** |
| BE_60_Cluster-3379.13951 | vinculin | vcl | 0.57* |
| AE_Unf_Cluster-5694.11445 | novel protein containing zona pellucida-like domains | ZP2 | 0.03* |
| AE_30_Cluster-7470.14901 | zona pellucida sperm-binding protein 3 isoform X1 | ZP3X1 | 0.13** |
| BE_O_Cluster-7693.32526 | fucolectin | fuc-l | 0.17** |
| BE_Unf_Cluster-8245.1416 | cystatin-B | cysB | 0.13** |
| BE_O_Cluster-7693.21417 | mucin-17 | muc-17 | 0.57** |

Note: ^a^ the fold changes are indicated as compared with adhesive eggs at 1h postfertilization (AE-1h-FE). Values >1 indicate up-regulation, and values <1 indicate down-regulation. Proteins with fold difference >1.5 or <0.67 were considered significantly altered, which are indicated with * (*p* < 0.05) or ** (*p* < 0.01).

**Table S5. Summary of sequencing of *C. alburnus* transcriptome.**

| **Sample** | **Raw Reads** | **Clean reads** | **Clean bases** | **Error (%)** | **Q20(%)** | **Q30(%)** | **GC (%)** |
| --- | --- | --- | --- | --- | --- | --- | --- |
| AE_Unf_1 | 57876388 | 55662070 | 8.35G | 0.01 | 97.36 | 93.44 | 41.91 |
| AE_Unf_2 | 64463426 | 61987784 | 9.3G | 0.01 | 97.42 | 93.40 | 45.72 |
| AE_Unf_3 | 58380824 | 56618442 | 8.49G | 0.01 | 97.47 | 93.46 | 46.44 |
| AE_0_1 | 63269182 | 61037504 | 9.16G | 0.02 | 96.90 | 92.39 | 47.28 |
| AE_0_2 | 57863304 | 55066106 | 8.26G | 0.01 | 97.58 | 93.66 | 47.16 |
| AE_0_3 | 55572814 | 52971678 | 7.95G | 0.01 | 97.37 | 93.21 | 45.87 |
| AE_30_1 | 50574866 | 48381796 | 7.26G | 0.01 | 97.22 | 92.93 | 45.86 |
| AE_30_2 | 54944248 | 52301318 | 7.85G | 0.02 | 97.12 | 92.78 | 44.91 |
| AE_30_3 | 59740432 | 56515578 | 8.48G | 0.01 | 97.41 | 93.30 | 45.86 |
| AE_60_1 | 58066328 | 56707340 | 8.51G | 0.02 | 96.84 | 92.37 | 46.55 |
| AE_60_2 | 70765652 | 68900288 | 10.34G | 0.02 | 96.99 | 92.62 | 46.86 |
| AE_60_3 | 65417080 | 62125112 | 9.32G | 0.02 | 96.91 | 92.51 | 44.93 |
| BE_Unf_1 | 40860796 | 39364826 | 5.9G | 0.01 | 97.84 | 94.51 | 44.53 |
| BE_Unf_2 | 47994728 | 46640396 | 7G | 0.01 | 98.38 | 95.75 | 46.50 |
| BE_Unf_3 | 50378834 | 48100598 | 7.22G | 0.01 | 97.66 | 94.05 | 47.02 |
| BE_0_1 | 57739178 | 54155744 | 8.12G | 0.01 | 97.72 | 94.25 | 44.14 |
| BE_0_2 | 57393360 | 54085020 | 8.11G | 0.01 | 97.66 | 94.17 | 44.04 |
| BE_0_3 | 63542604 | 60564584 | 9.08G | 0.01 | 97.60 | 94.09 | 43.33 |
| BE_30_1 | 51132008 | 49573302 | 7.44G | 0.01 | 98.34 | 95.74 | 44.07 |
| BE_30_2 | 52779336 | 51051930 | 7.66G | 0.01 | 98.33 | 95.73 | 44.22 |
| BE_30_3 | 49548660 | 47950460 | 7.19G | 0.01 | 98.31 | 95.67 | 44.83 |
| BE_60_1 | 51006840 | 49406208 | 7.41G | 0.01 | 98.35 | 95.74 | 44.35 |
| BE_60_2 | 47891606 | 46522232 | 6.98G | 0.01 | 98.31 | 95.62 | 46.32 |
| BE_60_3 | 76118308 | 72221238 | 10.83G | 0.01 | 97.63 | 94.16 | 42.58 |
| AE_O_1 | 48141188 | 46309736 | 6.95G | 0.01 | 97.84 | 94.61 | 47.02 |
| AE_O_2 | 44486962 | 41454442 | 6.22G | 0.02 | 94.84 | 88.38 | 47.67 |
| AE_O_3 | 42914658 | 40844984 | 6.13G | 0.02 | 95.96 | 90.48 | 47.76 |
| BE_O_1 | 84928468 | 82462324 | 12.37G | 0.01 | 97.52 | 94.01 | 48.74 |
| BE_O_2 | 56771318 | 55056244 | 8.26G | 0.01 | 97.39 | 93.69 | 48.64 |
| BE_O_3 | 56640088 | 54713060 | 8.21G | 0.01 | 97.50 | 93.91 | 48.86 |

Note: Q20, Q30, the percentage of bases with Phred (-10log10(e)) values greater than 20 or 30 in the total base; N50, unigene length-weighted median; GC, the percentage of the total number of base G and C in the total base number.

**Table S6. Transcript levels for enzymes involved in biosynthesis of heparan sulphate and chondroitin/dermatan sulphates in the ovary in two ecotypes of *C. alburnus*.**

| **Gene accession** | **Gene description** | **Abbreviation** | **Log_2_Fold change^a^**  **AE-O /BE-O** |
| --- | --- | --- | --- |
| i1_LQ_AE_c103260/f1p3/1619 | β-1,4-galactosyltransferase 7 | B4GALT7 | 1.26** |
| i1_LQ_AE_c65112/f1p1/1467 | β-1,3-galactosyltransferase 6 | B3GALT6 | 8.62** |
| i5_LQ_BE_c10551/f1p3/5178 | Exostosin-like 3 | EXTL3 | 2.56** |
| i1_LQ_BE_c75395/f1p24/1524 | Exostosin-1b | EXT1 | -7.74** |
| i1_LQ_BE_c5444/f1p4/1614 | Exostosin-2 | EXT2 | 8.77** |
| i2_HQ_AE_c35424/f2p3/2443 | Heparan sulfate N-deacetylase/N-sulfotransferase | NDST | 0.74** |
| i2_LQ_AE_c10518/f1p12/2167 | Heparan sulphate glucosamine 3-O-sulfotransferase 1 | HS3ST1 | 3.02** |
| i2_LQ_BE_c48202/f1p0/2030 | Chondroitin sulphate N-acetylgalactosaminyltransferase 1 | CSGALNACT1 | 2.04** |
| i2_LQ_BE_c44912/f1p0/2595 | Chondroitin sulphate N-acetylgalactosaminyltransferase 2 | CSGALNACT2 | -0.82** |
| i3_LQ_AE_c18465/f1p5/3851 | Chondroitin sulphate synthase 1 | CHSY1 | 8.13** |
| i3_LQ_BE_c8491/f1p0/3702 | Chondroitin polymerizing factor a | CHPF | 5.94** |
| i1_LQ_BE_c59173/f1p2/1619 | Uronyl 2-sulfotransferase | UST | 8.17** |
| i3_LQ_BE_c8214/f1p3/3732 | Dermatan-sulphate epimerase | DSE | -0.49** |

Note: ^a^ the fold changes are indicated as compared with the *C. alburnus*-B ovary (BE-O). Values >1 indicate up-regulation, and values <1 indicate down-regulation. Transcript levels with fold difference >1.2 or <0.83 were considered significantly altered, which are indicated with ** (*p* < 0.01).
